# Supplementary material for: Informing the development of a standardised approach to measure antibiotic use in secondary care: a systematic review protocol
Source: BMJ Open. 2019 May 14;9(5):e026792. doi: 10.1136/bmjopen-2018-026792 (PMC6530450; doi:10.1136/bmjopen-2018-026792)
Supplement: Supplementary data [file bmjopen-2018-026792supp001.pdf]

**SUPPLEMENTARY MATERIALS**

**Table S1.** ***World Bank Country Lending Groups Classification: High-Income Economies ($12,056 GNI per Capita or more)***

Accessed on: 23.08.18. Available at: https://datahelpdesk.worldbank.org/knowledgebase/articles/906519-world-bank-country-and-lending-groups

| Countries | | | | |
|---|---|---|---|---|
| Andorra | Germany | Oman | Antigua and Barbuda | Gibraltar |
| Palau | Argentina | Greece | Panama | Aruba |
| Greenland | Poland | Australia | Guam | Portugal |
| Austria | Hong Kong SAR, China | Puerto Rico | The Bahamas | Hungary |
| Qatar | Bahrain | Iceland | San Morino | Barbados |
| Ireland | Saudi Arabia | Belgium | Isle of Man | Seychelles |
| Bermuda | Israel | Singapore | British Virgin Islands | Italy |
| Sint Maarten (Dutch) | Brunei Darussalam | Japan | Slovak Republic | Canada |
| Korea Rep. | Slovenia | Cayman Islands | Kuwait | Spain |
| Channel Islands | Latvia | St. Kitts and Nevis | Chile | Liechtenstein |
| St. Martin (French) | Croatia | Lithuania | Sweden | Curaçao |
| Luxembourg | Switzerland | Cyprus | Macao SAR, China | Taiwan, China |
| Czech Republic | Malta | Trinidad and Tobago | Denmark | Monaco |
| Turks and Caicos Islands | Estonia | Netherlands | United Arab Emirates | Faroe Islands |
| New Caledonia | United Kingdom | Finland | New Zealand | United States |
| France | Northern Mariana Islands | Uruguay | French Polynesia | Norway |
| Virgin Islands (U.S.) | | | | |

**Table S2.** ***Website Search List***

Expanded upon from:

Stanic Benic, M. *et al.* Metrics for quantifying antibiotic use in the hospital setting: results from a systematic review and international multidisciplinary consensus procedure. *J. Antimicrob. Chemother.* **73,** vi50-vi58 (2018).

| | Organisation | Website(s) |
|---|---|---|
| 1 | Africa CDC: African Union | https://au.int/en/africacdc |
| 2 | Agency for Healthcare Research and Quality | www.ahrq.gov/ |
| 3 | Antibiotic Action | http://antibiotic-action.com/ |
| 4 | Alliance for the Prudent Use of Antibiotics (APUA) | www.apua.org/ |
| 5 | Australian Commission on Safety and Quality in Health Care \| Antimicrobial Use and Resistance in Australia (AURA) | https://www.safetyandquality.gov.au/antimicrobial-use-and-resistance-in-australia/ |
| 6 | Australian Government Department of Health | www.health.gov.au/ |
| 7 | British Society for Antimicrobial Chemotherapy | http://www.bsac.org.uk/ |
| 8 | British Infection Association | https://www.britishinfection.org/ |
| 9 | Center for Disease Dynamics, Economics & Policy (CDDEP) | https://www.cddep.org/<br>https://resistancemap.cddep.org/AntibioticUse.php |
| 10 | Canadian Foundation for Healthcare Improvement | https://www.cfhi-fcass.ca/ |
| 11 | Centers for Disease Control and Prevention (CDC) | https://www.cdc.gov/ |
| 12 | Care Quality Commission | https://www.cqc.org.uk/ |
| 13 | European Centre for Disease Control and Prevention (ECDC) | https://ecdc.europa.eu/en/home |
| 14 | European Society of Clinical Microbiology and Infectious Diseases (ESCMID) | https://www.escmid.org/ |
| 15 | Healthcare Infection Association | https://www.britishinfection.org/ |
| 16 | Institute for Healthcare Improvement: Australian Council for Safety and Quality Health Care | http://www.ihi.org/resources/Pages/OtherWebsites/AustralianCouncilforSafetyandQualityinHealthCare.aspx |
| 17 | International Society of Chemotherapy Infection and Cancer (ISC) | www.ischemo.org/ |
| 18 | International Society of Infectious Diseases | www.isid.org/ |
| 19 | National Institute for Health and Care Excellence (NICE) | https://www.nice.org.uk/ |
| 20 | National Quality Measures Clearinghouse | https://www.qualitymeasures.ahrq.gov/ |
| 21 | NHS Improvement | https://improvement.nhs.uk/ |
| 22 | Public Health Agency of Canada | www.publichealth.gc.ca/ |

| | | |
|---|---|---|
| 23 | Public Health England | https://www.gov.uk/government/organisations/public-health-england<br>https://fingertips.phe.org.uk/profile/amr-local-indicators |
| 24 | Public Health Surveillance: New Zealand Ministry of Health & Institute of Environmental Science Research Ltd (ESR) | https://surv.esr.cri.nz/index.php |
| 25 | RAND Corporation | www.rand.org/ |
| 26 | ReAct group | www.reactgroup.org/ |
| 27 | Swedish Strategic Programme against Antibiotic Resistance (STRAMA) | www.strama.se |
| 28 | Transatlantic Task Force on Antimicrobial Resistance (TATFAR) | http://www.cdc.gov/drugresistance/tatfar/ |
| 29 | US Food and Drug Administration | www.fda.gov/ |
| 30 | World Health Organization | www.who.int/<br>http://www.afro.who.int/<br>https://www.paho.org/hq/<br>http://www.searo.who.int/en/<br>http://www.euro.who.int/en/home<br>http://www.emro.who.int/index.html<br>http://www.wpro.who.int/en/ |
| 31 | Scottish Antimicrobial Prescribing Group | http://www.isdscotland.org/Health-Topics/Prescribing-and-Medicines/SAPG/ |
