# Supplementary material for: Informing the development of a standardised approach to measure antibiotic use in secondary care: a systematic review protocol
Source: BMJ Open. 2019 May 14;9(5):e026792. doi: 10.1136/bmjopen-2018-026792 (PMC6530450; doi:10.1136/bmjopen-2018-026792)
Supplement: Author's manuscript [file bmjopen-2018-026792.draft_revisions.pdf]

BMJ Open

BMJ Open is committed to open peer review. As part of this commitment we make the peer review history of every article we publish publicly available.

When an article is published we post the peer reviewers' comments and the authors' responses online. We also post the versions of the paper that were used during peer review. These are the versions that the peer review comments apply to.

The versions of the paper that follow are the versions that were submitted during the peer review process. They are not the versions of record or the final published versions. They should not be cited or distributed as the published version of this manuscript.

BMJ Open is an open access journal and the full, final, typeset and author-corrected version of record of the manuscript is available on our site with no access controls, subscription charges or pay-per-view fees (http://bmjopen.bmj.com).

If you have any questions on BMJ Open's open peer review process please email info.bmjopen@bmj.com

BMJ Open

HOW CAN METRICS BE USED TO ROUTINELY MONITOR ANTIBIOTIC USAGE AND DEFINE (IN)APPROPRIATE ANTIBIOTIC USAGE IN SECONDARY CARE? A SYSTEMATIC REVIEW PROTOCOL

Journal:	*BMJ Open*
Manuscript ID	bmjopen-2018-026792
Article Type:	Protocol
Date Submitted by the Author:	19-Sep-2018
Complete List of Authors:	Patel, Selina; University College London, Institute of Health Informatics<br>Jhass, Arnoupe; University College London, Department of Primary Care and Population Health, Institute of Epidemiology and Health Care<br>Hopkins, Susan; Public Health England<br>Shallcross, Laura; University College London, Institute of Health Informatics
Keywords:	antibiotic resistance, antibiotic usage, secondary care, high resource

SCHOLARONE™
Manuscripts

TITLE: HOW CAN METRICS BE USED TO ROUTINELY MONITOR ANTIBIOTIC USAGE AND DEFINE (IN)APPROPRIATE ANTIBIOTIC USAGE IN SECONDARY CARE? A SYSTEMATIC REVIEW PROTOCOL

CORRESPONDING AUTHOR: Selina Patel

Email: selina.patel.17@ucl.ac.uk

Address: 222 Euston Road, London, NW1 2DA

AUTHORS: Selina Patel[1], Arnoupe Jhass[2], Susan Hopkins[3], Laura Shallcross[1]

WORD COUNT: 3735

KEYWORDS: antibiotic resistance; antibiotic usage; secondary care; high-resource

ABSTRACT

Introduction

Global ambitions have been set by the World Health Organization and national governments to improve the availability of data around antibiotic usage. In secondary care, the Global Point Prevalence Survey conducted in 2015 estimates that 34.4% of hospital inpatients globally are receiving at least one antimicrobial. In this setting patients have a high risk of acquiring and transmitting drug resistant infections. Antimicrobial stewardship to optimize antibiotic usage in secondary care can reduce this risk. However, differences in the availability of data on antibiotic usage in this context make it difficult to develop a consensus of how to monitor antibiotic prescribing patterns in secondary care. This matters because effective surveillance underpins the development of evidence-based antimicrobial stewardship initiatives.

[1] Public Health Data Science, Institute of Health Informatics, University College London
[2] Department of Primary Care & Population Health, Institute of Epidemiology and Health Care, University College London
[3] Public Health England

For peer review only

Methods and Analysis

We will search Medline (Ovid), Embase (Ovid), CINAHL, Cochrane Central Register of Controlled Trials and websites of key organisations for published reports where an attempt to measure antibiotic usage among adult in-patients in high-income hospital settings has been made. Two independent reviewers will screen the studies for eligibility, extract data and assess the study quality and risk of bias. An adaptation of the APEASE framework will be used to consider the practicality of implementing different approaches to measuring antibiotic usage in secondary care settings. A description and comparison of definitions and estimates of (in)appropriate antibiotic usage will also be carried out.

Ethics and Dissemination

Ethical approval is not required for this study as no primary data will be collected. The results of this review will be published in relevant peer reviewed journals and presented at relevant conferences or meetings where possible.

PROSPERO Registration Number

CRD42018103375

ARTICLE SUMMARY

Strengths and limitations of this study

This study protocol follows the recommendations outline in the Preferred Reporting Items for Systematic Review and Meta-analysis Protocols.

This protocol has been registered on the International Prospective Register of Systematic Reviews.

The selection of studies, data extraction and study quality assessments will be conducted by two independent reviewers.

For peer review only

Introduction

It is estimated that by the year 2050, 10 million deaths per year will be attributable to antimicrobial resistance (AMR).[1] The mutation and evolution of bacteria in the presence of antibiotics to resist antibiotic action is a natural process in the environment. However, the introduction of antibiotics to treat disease in humans exerts an artificially enhanced selection pressure that favors the selection of drug resistant strains of bacteria. There is good evidence from ecological studies that at the population level higher rates of antibiotic resistance are associated with high levels of antibiotic consumption.[2] There are increasing data to indicate that antibiotic exposure promotes resistance at the individual level also.[3] One way in which to combat this global public health threat is through antimicrobial stewardship (AMS) which optimizes the use of antimicrobials to safely reduce the total quantity of antimicrobials consumed. The surveillance of antimicrobial consumption is the foundation of monitoring the impact of any antimicrobial stewardship intervention to control the emergence and spread of AMR. This is recognized by a global focus on implementing antimicrobial stewardship (AMS) programmes which monitor the consumption of antibiotics.[4] Government initiatives are supporting this through AMR National Action Plans and activities such as the 'Fleming Fund', which aim to provide better quality data on prescribing and resistance by building surveillance capacity in developing countries.[5] However, despite this activity there is still a lack of consensus on how to interrogate antibiotic usage data to measure patterns of antibiotic prescribing, particularly in the secondary care setting.[6]

To give some examples, in Australia, The National Antimicrobial Utilization Surveillance Programme (NAUSP) collects data through a voluntary surveillance programme which is based on hospitals submitting data to the programme. The report provides limited information about stewardship prescribing patterns and focuses on quantities of antimicrobials prescribed by type per 1,000 occupied bed days.[7,8] The National Antimicrobial Prescribing Survey (NAPS) measures beyond this to quantify "appropriateness" of antibiotic prescribing by auditing a sample of hospital prescriptions. This collects data on some objective measures such as the proportion of prescriptions which document a stop or review

For peer review only

date, as well as some subjective measures such as the appropriateness of the prescription. These data are reported at a national and regional level, however the data may not be fully representative as it is conducted through a non-randomized voluntary survey.[9,10] In the UK, the Commissioning for Quality and Innovation (CQUIN) goes some way towards monitoring antibiotic stewardship in NHS England trusts aggregated at hospital-level through financially incentivizing stewardship behaviours outlined in the national '*Start Smart, Then Focus*' toolkit for antimicrobial prescribing in secondary care, in particular among sepsis patients. In order to measure patterns of antibiotic usage associated with stewardship in secondary care beyond counting quantities used by type of antibiotic, local audits are conducted in hospitals on a sample of prescriptions.[11,12] However, this does not capture prescriber-level stewardship behaviours and so does not enable hospitals to identify where antibiotic stewardship is not being carried out and ascertain if it could be appropriate to target intervention resources to areas where they could have the greatest impact.

The NAUSP, NAPS and CQUIN initiatives feedback the data provided aggregated at the hospital or national level, similar to *OpenPrescribing.net* for primary care in the UK.[13] However, secondary care is a dynamic setting and many different clinicians across specialties and a spectrum of seniority will be involved in the prescribing process at different stages of: antibiotic initiation, treatment selection, clinical review, de-escalation and cessation of treatment. This is different to the primary healthcare setting where patients usually have a single consultation which may result in an antibiotic prescription. This makes understanding patterns of prescribing within hospitals more difficult to map and highlights the usefulness of using patient-level prescribing data linked to the prescriber, or the specialty of the prescriber, to monitor patterns of antimicrobial usage and stewardship as patients move through the hospital. The widespread establishment of electronic prescribing systems in the USA and the proposed investments in electronic prescribing systems in countries such as the UK and Australia, provides a potential opportunity to digitize systems to monitor antimicrobial usage and stewardship in secondary care, and capture patient-level prescribing data to understand patterns of prescribing.[14,15] This would enable descriptions of patterns

For peer review only

of prescribing within hospitals to better understand the drivers of appropriate and inappropriate antibiotic usage in secondary care. This could support better targeting of stewardship efforts to those areas where they can have the greatest impact and provide the means to measure the impact of novel stewardship interventions.

At present, high income secondary care settings have different levels of data and resources available to monitor antimicrobial usage and stewardship; from aggregate data on prescribing to integrated electronic health record and patient-level prescribing data. As high-income settings answer the WHO call to increase the availability of antimicrobial usage data, there is a need to reach a consensus on how to interrogate this data and be able to draw comparisons between hospital sites.[4] This must take account of factors such as the availability of different data sources, infrastructure, digital maturity and the availability of trained staff with the skills to interrogate these datasets. To establish a universal surveillance methodology which is applicable to different data sources at different hospital sites means that high-level metrics applied to aggregate prescribing data must also be applicable to patient-level electronic prescribing data which may be taken from a database which records daily dose administration with no record of complete prescriptions. The consequence of variation in prescribing processes and systems between hospitals and variation in the availability of resources when attempting to monitor patterns of prescribing in secondary care is a lack of consensus and previous attempts to establish a conventional approach have failed to address all of these issues.

Recently, two studies attempted to establish a consensus on quality and quantity indicators for measuring antibiotic use in secondary care using systematic review and RAND-modified Delphi methods.[16,17] However, neither study considered differences in the availability of data and resources between hospital settings, or how these approaches might be applied to patient-level data to explore patterns of prescribing within a hospital. Applying metrics for antibiotic usage to patient-level data to explore intra-hospital patterns of prescribing requires a clear methodology for the attribution of the prescribing pathway to wards, specialties or consultant teams. To develop robust systems to monitor

For peer review only

antibiotic usage in secondary care requires the development of simple measures which enable comparisons of antibiotic usage over time and across specialties. Critically, these measures must be meaningful for clinicians, and be based on data that are routinely available, since few hospitals have the resources to conduct regular audits of antimicrobial use.

This review will therefore study attempts to quantify total systemic antibiotic usage, appropriate antibiotic usage and inappropriate usage in high income settings in secondary care. This review will provide a summary of the ways in which antibiotic usage can be measured alongside a narrative about the practicality of employing these metrics in secondary care based on an adaptation of the APEASE framework.

OBJECTIVES

The aim of this review is to provide an outline of the methodologies to quantify antibiotic usage among in-patients in secondary care. The objectives are:

- To identify the data sources and metrics which have most frequently been used to represent antibiotic usage in secondary care.
- To assess the suitability of metrics of antibiotic usage for use in routine surveillance of antibiotic usage.
- To document the variation in definitions and estimates of appropriate and inappropriate antibiotic usage.

METHODS

The following methods are based on the framework provided by the 'Preferred Reporting Items for Systematic review and Meta-Analysis Protocols (PRISMA-P) checklist.[18]

Eligibility Criteria

For peer review only

Types of Studies

The article must make an attempt(s) to quantify antibiotic usage in at least 100 adult patients (>17 years old) among in-patients in secondary care. Where the number of patients is not detailed, the article will be excluded from the review. Articles which focus on antibiotic usage among individuals with specific conditions or specific demographic groups will be included. The nature of these criteria is such that we would expect a range of study designs which are based on both observation and intervention, and which have attempted to estimate antibiotic usage in secondary care for a variety of purposes.

This review will not include trials of antibiotic therapies in secondary care or reviews of the literature. Studies which are based exclusively on mathematically modelled predictions of the inclusion criteria data will also be excluded from the study. Those articles which exclusively describe a methodology for measuring antibiotic usage and which do not attempt to implement it to estimate antibiotic usage will be excluded. Articles which are not written in English-language and which are based on antibiotic usage data related to non-human participants will also be excluded.

Types of Participants

The article must be research based on dispensing, prescribing, survey/audit or sales data collected from the in-patient secondary care setting. It is expected that hospitals in high-income settings will be very different to those in low-resource settings in both the availability of data and resources available for stewardship, therefore this review will focus on hospitals in high-income countries as classified by the World Bank only (**Table S1**).[19] When the country is not classified in the World Bank lending groups, the article will be included if the gross national income (GNI) per capita is 12,056 USD or more. Articles must attempt to quantify antibiotic usage among adult patients (>17-years old); those articles which exclusively document usage among non-adult patients (under 18-years old) will be excluded.

Types of Interventions

For peer review only - http://bmjopen.bmj.com/site/about/guidelines.xhtml

This review will consider all attempts to measure antibiotic usage among at least 100 patients in the hospital in-patient setting.

Types of Comparators

There are no comparison groups for this review as it is an exploration to identify the methodologies of how antibiotic usage has been measured in secondary care, and how this has been used to define and estimate appropriate and inappropriate antibiotic usage.

Types of Outcome measures

Articles which report a measure of antibiotic usage in hospital will be included in this review.

Primary Outcomes

- Data source used to represent antibiotic usage.
- Metrics which attempt to quantify antibiotic usage.
- An analysis of the suitability of existing metrics of antibiotic usage for use in routine practice to monitor antibiotic usage based on an adaptation of the APEASE framework.[20]

Secondary Outcomes

- Quantitative definitions of appropriate and inappropriate antibiotic usage.
- Comparison of the definitions of appropriate and inappropriate prescribing in secondary care.
- Description of variations in estimates of appropriate and inappropriate prescribing in secondary care.

Information Sources

Electronic Searches

The following databases will be searched for relevant articles:

For peer review only

- Medline (Ovid);
- Embase (Ovid);
- CINAHL;
- Cochrane Central Register of Controlled Trials;

The search strategy will be developed in Medline and then adapted appropriately for use in each database and run between August 2018 and September 2018.

Website Searches

In order to capture reports from the grey literature, the websites of relevant organisations will be searched (**Table S2**). The list of relevant organisations was expanded from a list originally conceived by *Benic et. al* based on discussion between SP, AJ and LS.[21] This review will aim to consider the suitability of existing metrics of antibiotic usage in routine hospital surveillance of antibiotic usage; therefore it is essential that approaches to do this on a large scale such as through National Action Plans, which are often published in the grey literature, should be captured in our search.

Search Strategy

The provisional search strategy is adapted from the work of *Benic et. al* based on four concepts: antibiotics; utilisation; measure; hospital (**Table 1**).[21] The search strategy will be established in MEDLINE and then adapted to the other 3 databases included in the search. No restrictions will be placed on the search. The bibliographies of those articles selected for inclusion in the review will also be searched to capture any remaining articles not identified by the search.

Table 1. Medline (Ovid) provisional search terms.

Search Concept	Search Terms
**Antibiotic**	1. Anti-Bacterial Agents/ad, dt, sd, tu, th, ut [Administration & Dosage, Drug Therapy, Supply & Distribution, Therapeutic Use, Therapy, Utilization]

	2. Antibiotic Prophylaxis/ec, mt, sn, td, ut [Economics, Methods, Statistics & Numerical Data, Trends, Utilization]
	3. (anti?biotic? or anti?microbial? or anti?bacterial?).ab,ti.
	4. 1 or 2 or 3
**Utilisation**	5. Drug Prescriptions/
	6. Drug Utilization/
	7. "Drug Utilization Review"/cl, ec, mt, st, sn, td, ut [Classification, Economics, Methods, Standards, Statistics & Numerical Data, Trends, Utilization]
	8. ((anti?biotic? or anti?microbial?) adj3 (prescri* or consumption or utili?ation or usage or "use" or dispens* or sale?)).ab,ti.
	9. 5 or 6 or 7 or 8
**Measurement**	10. BENCHMARKING/cl, ec, mt, st, sn, td, ut [Classification, Economics, Methods, Standards, Statistics & Numerical Data, Trends, Utilization]
	11. (intervention adj5 (prescri* or stewardship or "use" or utili?ation or usage or consumption)).ab,ti
	12. ((anti?biotic? or anti?microbial?) adj4 (estimat* or quanitf* or metric? or monitor* or surveillance or prevalence or survey or audit)).ab,ti.
	13. (electronic prescri* or e?prescri*).ab,ti.
	14. 10 or 11 or 12 or 13
**Secondary Care**	15. Secondary Care/
	16. Hospitals/
	17. hospital*.ab,ti.
	18. 15 or 16 or 17
	19. 4 and 9 and 14 and 18

For peer review only

Study Records

Data Management

The articles will be uploaded from the relevant database to *Mendeley* reference manager software. The reports will be exported to the web-based, *EPPI Reviewer 4* systematic review software which will be used to remove duplicate articles. Each stage of the screening process will be carried out in *EPPI Reviewer 4* using a standardised form of article eligibility. The online form for article eligibility will be piloted before the start of the screening process and amended as required to ensure it is fit for purpose. The data will be extracted and entered into a standardised, piloted, pre-created online data extraction form in *EPPI Reviewer 4* in duplicate by two independent reviewers (SP and AJ).

Selection Process

The unique articles captured by the search will undergo a title pre-screening by a single reviewer (SP). The remaining articles will then be screened based on title and abstract by two independent reviewers in duplicate (SP and AJ)); when study eligibility is not mutually agreed upon or is ambiguous from the title and abstract only, the article will be admitted into the full-text review stage. The selected articles will then finally go through full-text review by two independent reviewers in duplicate (SP and AJ). Any disagreements between authors at any stage will be resolved through a third reviewer (LS). The reasons for the exclusion of these studies from this review will be documented for reference and the study selection process will be presented as a PRISMA flow diagram.[18]

Data Collection Process

The data will be collected in duplicate by two independent reviewers (SP and AJ) from articles selected for inclusion in the review. The data will be entered into pre-created, piloted forms on EPPI Reviewer 4: systematic review software. Any disagreement will be resolved through consultation with a third author (LS).

For peer review only

Data Items

We will extract data on:

- **Study** (study design, country, population, service setting such as size of hospital, objective).
- **Data type used to describe antibiotic usage** (data source, method of collection, frequency of the data collection, who collected the data, level of detail in the data and data type).
- **Metrics used to measure antibiotic usage** (numerator, denominator, units (such as per admission), level at which the metric is presented (such as by specialty) and what the metric is currently used for (such as national or hospital-level surveillance)).
- **Criteria used to define appropriate and inappropriate antibiotic usage**.
- **Data type used to quantify appropriate and inappropriate antibiotic usage** (data source, method of collection, level of detail in the data and data type).
- **Quantitative estimates of appropriate and inappropriate antibiotic usage** (numerator, denominator, units (such as per admission), level at which the metric is presented (such as by specialty) and what the metric is currently used for (such as national or hospital-level surveillance)).
- **Study quality and selection bias of those studies which provide an estimate of appropriate and inappropriate antibiotic usage** (outlined below under *Assessment of Risk of Bias*).

Outcomes Prioritisation

The main outcome of this review is a description of the data sources and metrics which have been used to measure antibiotic usage and to define appropriate and inappropriate antibiotic usage in hospitals. This will include a description of the teams most frequently involved in this type of work in order to identify those groups who are often facilitators of this surveillance.

For peer review only

This will then be used to consider each metric within an adaptation of the APEASE framework to assess the practicality of each metric for use in routine surveillance of antibiotic usage; this method is detailed in '*Data synthesis'*.[20] This aims to provide the detail to inform the methodology of sustainable antibiotic usage surveillance programmes. We will also document if it is stated that the metric is currently being routinely used for any purpose such as national level surveillance. This will identify reasons why antibiotic usage surveillance is already being implemented routinely and will highlight programmes where existing infrastructure may be extended for use to routinely monitor antibiotic usage within hospitals. If the metric has been used to define and estimate (in)appropriate antibiotic usage, this will be used to explore the variation in the definitions and estimates of inappropriate antibiotic usage in hospitals. It is anticipated that such estimations will have been made using a multitude of methodologies and measures. The extent to which quantitative estimates of inappropriate prescribing can be directly compared may therefore be limited.

Assessment of Risk of Bias in Individual Studies

This review is a descriptive study to consider metrics which have been used to quantify antibiotic usage in the in-patient secondary care setting, and the suitability of their use for routine monitoring of antibiotic usage. It is therefore necessary for all metrics to have been utilized in the secondary care setting to capture the antibiotic usage data for at least 100 patients. This is to ensure some level of demonstration of the feasibility of implementing the measures. Where the number of patients for which data has been captured using these metrics has not been detailed, the articles will be included in the review. For these descriptive purposes, it is not appropriate to use a risk of bias framework.

For studies included in the full-text review stage and which attempt to make a quantitative estimate of appropriate or inappropriate antibiotic usage in the secondary care setting, the Newcastle-Ottawa scale to assess risk of bias will be used to determine the inclusion of the estimate in the review.[22]

Data synthesis

For peer review only

The data will undergo a descriptive synthesis to provide insight into the methodologies to implement antibiotic usage metrics in secondary care. We will record the frequency of use of different data sources by level (individual to national) and by source (such as electronic prescribing or audits). This will examine the type of data which is currently being used to monitor antibiotic usage and consequently the level detail in which we are able to examine prescribing currently. We will also identify the frequency of use of each metric to measure: volume, type, duration, stewardship activity and prevalence/incidence of antibiotic use. As well as identify the level at which these metrics are most frequently presented such as by specialty or nationally. The teams involved in these studies to collect and analyse the data will also be recorded.

An adaptation of the APEASE framework will be used to assess the feasibility of metrics for use in routine monitoring of antibiotic usage. The APEASE framework was designed to aid the context-specific design and selection of interventions to promote health behaviour change.[20] The framework acronym is elaborated to: a – affordability; p – practicability; e – effectiveness and cost-effectiveness; a – acceptability; s – side-effects/safety; e – equity. This original framework will be adapted to inform the establishment of a consensus to monitor antibiotic usage in the high-income secondary care setting [**Table 2**].

Table 2. An adaptation of the APEASE framework to assess the feasibility of metrics for use in routine surveillance.

**A**	[Affordability] Resources required to sustainably implement the metric. For example, person time to audit / new system roll-out.
**P**	[Practicability] Can the metric be delivered? Is there sustainable access to data and resources?
**E**	[Effectiveness] Does the metric represent the data appropriately? Cost-effectiveness?
**A**	[Acceptability] Does it represent relevant data to monitor antibiotic usage and appropriateness of antibiotic usage?

For peer review only

**S**	[Side-effects] Bias of metrics
**E**	[Equity] Will the metric adequately highlight disparities and meaningfully inform the design of interventions?

This analysis can then be used to inform recommendations based on the available literature regarding a sustainable, conventional strategy to monitor antibiotic usage and the resources which would be required to practically implement such a surveillance strategy.

The variation between comparable hospital-level estimates of (in)appropriate antibiotic usage will be described using a funnel plot and 95% confidence intervals.

Meta-bias(es)

The systematic review will be conducted in line with this protocol and any aspects of the method which deviate from the original protocol will be reported in the final review. Only articles written in English-language will be included in this review; this is due to the language spoken by the authors.

Confidence in Cumulative Evidence

This is a descriptive review to consider the methodologies which have been used to measure antibiotic usage and to define appropriate and inappropriate antibiotic prescribing in hospitals. It is therefore not necessary to consider the quality of quantitative evidence. The frequency of the availability of the data that this protocol outlines we hope to capture from the study will be presented as a narrative in the final review as part of a summary of the articles included in the study.

Patient and Public Involvement

Patients and members of the public were not involved in the development of this protocol.

Contributors

For peer review only

The study was conceived by SP, SH and LS. SP developed the eligibility criteria, search strategy, risk of bias assessment and the data extraction plans with guidance from LS. SP, AJ and LS developed the website search list. All authors contributed to and reviewed the final manuscript.

Acknowledgements

The authors acknowledge the valuable technical guidance offered by Suvi Härmälä in the construction of the protocol.

Funding

This review was funded by the Economic and Social Research Council (ESRC); grant number: ES/P000592/1 awarded to SP. The ESRC was not involved in the development of this protocol.

Patient Consent

Not required.

Ethics Approval

Ethical approval is not required for this study.

CONFLICTS OF INTEREST

The authors declare no conflicts of interest.

BIBLIOGRAPHY

1. O'Neil, J. *TACKLING DRUG-RESISTANT INFECTIONS GLOBALLY: FINAL REPORT AND RECOMMENDATIONS THE REVIEW ON ANTIMICROBIAL RESISTANCE*. (2016).

2. Goossens, H., Ferech, M., Vander Stichele, R. & Elseviers, M. Outpatient antibiotic use in Europe and association with resistance: a cross-national database study. *Lancet* **365,** 579–587 (2005).

For peer review only

3. Costelloe, C., Metcalfe, C., Lovering, A., Mant, D. & Hay, A. D. Effect of antibiotic prescribing in primary care on antimicrobial resistance in individual patients: systematic review and meta-analysis. *BMJ* **340,** c2096 (2010).

4. World Health Organization. *Global Action Plan on Antimicrobial Resistance*. (2015).

5. The Fleming Fund. Fleming Fund > Country Grants. Available at: http://www.flemingfund.org/Country-Grants. (Accessed: 20th August 2018)

6. Zanichelli, V. *et al.* Variation in antibiotic use among and within different settings: a systematic review. *J. Antimicrob. Chemother.* **73,** vi17-vi29 (2018).

7. Australian Commission on Safety and Quality in Health Care. Antimicrobial Use in Australian Hospitals (NAUSP) | Safety and Quality. Available at: https://www.safetyandquality.gov.au/antimicrobial-use-and-resistance-in-australia/nausp/?section=4. (Accessed: 22nd August 2018)

8. *Antimicrobial Use in Australian Hospitals 2016 annual report of the National Antimicrobial Utilisation Surveillance Program Published by the Australian Commission on Safety and Quality in Health Care*. (2018).

9. Australian Commission on Safety and Quality in Health Care. Appropriateness of antimicrobial use in Australian hospitals (NAPS) | Safety and Quality. Available at: https://www.safetyandquality.gov.au/antimicrobial-use-and-resistance-in-australia/naps-2/?section=4. (Accessed: 22nd August 2018)

10. Commission on Safety, A. & in Health Care, Q. *Antimicrobial prescribing practice in Australian hospitals*. (2016).

11. NHS England » Commissioning for Quality and Innovation (CQUIN) Guidance for 2017-2019.

For peer review only

Available at: https://www.england.nhs.uk/publication/commissioning-for-quality-and-innovation-cquin-guidance-for-2017-2019/. (Accessed: 24th August 2018)

12. Commissioning for Quality and Innovation. *CQUIN Indicator Specification Information on CQUIN*. (2017).

13. EBM DataLab & University of Oxford. Home | OpenPrescribing. Available at: https://openprescribing.net/. (Accessed: 13th August 2018)

14. Drug errors cause appalling harm and deaths, says Hunt - BBC News. Available at: https://www.bbc.co.uk/news/health-43161929. (Accessed: 14th August 2018)

15. Australian Government Department of Health. Improving Access to Medicines – ePrescribing for safer medicines. Available at: http://www.health.gov.au/internet/budget/publishing.nsf/Content/budget2018-factsheet14.htm. (Accessed: 24th August 2018)

16. Monnier, A. A. *et al.* Quality indicators for responsible antibiotic use in the inpatient setting: a systematic review followed by an international multidisciplinary consensus procedure. *J. Antimicrob. Chemother.* **73,** vi30-vi39 (2018).

17. Stanić Benić, M. *et al.* Metrics for quantifying antibiotic use in the hospital setting: results from a systematic review and international multidisciplinary consensus procedure. *J. Antimicrob. Chemother.* **73,** vi50-vi58 (2018).

18. Moher, D. *et. al*. Preferred Reporting Items for Systematic Review and Meta-Analysis Protocols (PRISMA-P) 2015 statement. Syst Rev. 2015;4(1):1.

19. Group, T. W. B. World Bank Country and Lending Groups. Available at: https://datahelpdesk.worldbank.org/knowledgebase/articles/906519-world-bank-country-and-

For peer review only

lending-groups.

20. Michie, S., Atkins, L. & West, R. *The Behaviour Change Wheel Book - A Guide To Designing Interventions*. (2014).

21. Stanic Benic, M. *et al.* Metrics for quantifying antibiotic use in the hospital setting: results from a systematic review and international multidisciplinary consensus procedure. *J. Antimicrob. Chemother.* **73,** vi50-vi58 (2018).

22. Wells, G. *et al.* The Newcastle-Ottawa Scale (NOS) for assessing the quality of nonrandomised studies in meta-analyses. Available at: http://www.ohri.ca/programs/clinical_epidemiology/oxford.asp. (Accessed: 31st July 2018)

For peer review only

SUPPLEMENTARY MATERIALS

Table S1. ***World Bank Country Lending Groups Classification: High-Income Economies ($12,056 GNI per Capita or more)***

Accessed on: 23.08.18. Available at: https://datahelpdesk.worldbank.org/knowledgebase/articles/906519-world-bank-country-and-lending-groups

**Countries**				
Andorra	Germany	Oman	Antigua and Barbuda	Gibraltar
Palau	Argentina	Greece	Panama	Aruba
Greenland	Poland	Australia	Guam	Portugal
Austria	Hong Kong SAR, China	Puerto Rico	The Bahamas	Hungary
Qatar	Bahrain	Iceland	San Morino	Barbados
Ireland	Saudi Arabia	Belgium	Isle of Man	Seychelles
Bermuda	Israel	Singapore	British Virgin Islands	Italy
Sint Maarten (Dutch)	Brunei Darussalam	Japan	Slovak Republic	Canada
Korea Rep.	Slovenia	Cayman Islands	Kuwait	Spain
Channel Islands	Latvia	St. Kitts and Nevis	Chile	Liechtenstein
St. Martin (French)	Croatia	Lithuania	Sweden	Curaçao
Luxembourg	Switzerland	Cyprus	Macao SAR, China	Taiwan, China
Czech Republic	Malta	Trinidad and Tobago	Denmark	Monaco
Turks and Caicos Islands	Estonia	Netherlands	United Arab Emirates	Faroe Islands
New Caledonia	United Kingdom	Finland	New Zealand	United States
France	Northern Mariana Islands	Uruguay	French Polynesia	Norway
Virgin Islands (U.S.)				

For peer review only

Table S2. ***Website Search List***

Expanded upon from:

Stanic Benic, M. *et al.* Metrics for quantifying antibiotic use in the hospital setting: results from a systematic review and international multidisciplinary consensus procedure. *J. Antimicrob. Chemother.* **73,** vi50-vi58 (2018).

	Organisation	Website(s)
1	Africa CDC: African Union	https://au.int/en/africacdc
2	Agency for Healthcare Research and Quality	www.ahrq.gov/
3	Antibiotic Action	http://antibiotic-action.com/
4	Alliance for the Prudent Use of Antibiotics (APUA)	www.apua.org/
5	Australian Government Department of Health	www.health.gov.au/
6	British Society for Antimicrobial Chemotherapy	http://www.bsac.org.uk/
7	British Infection Association	https://www.britishinfection.org/
8	Center for Disease Dynamics, Economics & Policy (CDDEP)	https://www.cddep.org/ https://resistancemap.cddep.org/AntibioticUse.php
9	Canadian Foundation for Healthcare Improvement	https://www.cfhi-fcass.ca/
10	Centers for Disease Control and Prevention (CDC)	https://www.cdc.gov/
11	Care Quality Commission	https://www.cqc.org.uk/
12	European Centre for Disease Control and Prevention (ECDC)	https://ecdc.europa.eu/en/home
13	European Society of Clinical Microbiology and Infectious Diseases (ESCMID)	https://www.escmid.org/
14	Healthcare Infection Association	https://www.britishinfection.org/
15	Institute for Healthcare Improvement: Australian Council for Safety and Quality Health Care	http://www.ihi.org/resources/Pages/OtherWebsites/AustralianCouncilforSafetyandQualityinHealthCare.aspx
18	International Society of Chemotherapy Infection and Cancer (ISC)	www.ischemo.org/
19	International Society of Infectious Diseases	www.isid.org/
20	National Institute for Health and Care Excellence (NICE)	https://www.nice.org.uk/
21	National Quality Measures Clearinghouse	https://www.qualitymeasures.ahrq.gov/
22	NHS Improvement	https://improvement.nhs.uk/
23	Public Health Agency of Canada	www.publichealth.gc.ca/
24	Public Health England	https://www.gov.uk/government/organisations/public-health-england https://fingertips.phe.org.uk/profile/amr-

For peer review only

		local-indicators
25	Public Health Surveillance: New Zealand Ministry of Health & Institute of Environmental Science Research Ltd (ESR)	https://surv.esr.cri.nz/index.php
26	RAND Corporation	www.rand.org/
27	ReAct group	www.reactgroup.org/
28	Swedish Strategic Programme against Antibiotic Resistance (STRAMA)	www.strama.se
29	Transatlantic Task Force on Antimicrobial Resistance (TATFAR)	http://www.cdc.gov/drugresistance/tatfar/
30	US Food and Drug Administration	www.fda.gov/
31	World Health Organization	www.who.int/<br>http://www.afro.who.int/<br>https://www.paho.org/hq/<br>http://www.searo.who.int/en/<br>http://www.euro.who.int/en/home<br><br>http://www.emro.who.int/index.html<br>http://www.wpro.who.int/en/
32	Scottish Antimicrobial Prescribing Group	http://www.isdscotland.org/Health-Topics/Prescribing-and-Medicines/SAPG/

For peer review only

Reporting checklist for protocol of a systematic review.

Based on the PRISMA-P guidelines.

Instructions to authors

Complete this checklist by entering the page numbers from your manuscript where readers will find each of the items listed below.

Your article may not currently address all the items on the checklist. Please modify your text to include the missing information. If you are certain that an item does not apply, please write "n/a" and provide a short explanation.

Upload your completed checklist as an extra file when you submit to a journal.

In your methods section, say that you used the PRISMA-P reporting guidelines, and cite them as:

Moher D, Shamseer L, Clarke M, Ghersi D, Liberati A, Petticrew M, Shekelle P, Stewart LA. Preferred Reporting Items for Systematic Review and Meta-Analysis Protocols (PRISMA-P) 2015 statement. Syst Rev. 2015;4(1):1.

		Reporting Item	Page Number
Identification	#1a	Identify the report as a protocol of a systematic review	1
Update	#1b	If the protocol is for an update of a previous systematic review, identify as such	N/A
	#2	If registered, provide the name of the registry (such as PROSPERO) and registration number	2
Contact	#3a	Provide name, institutional affiliation, e-mail address of all protocol authors; provide physical mailing address of corresponding author	1
Contribution	#3b	Describe contributions of protocol authors and identify the guarantor of the review	1
	#4	If the protocol represents an amendment of a previously completed or published protocol, identify as such and list changes; otherwise, state plan for documenting important	15

For peer review only

		protocol amendments	
Sources	#5a	Indicate sources of financial or other support for the review	16
Sponsor	#5b	Provide name for the review funder and / or sponsor	16
Role of sponsor or funder	#5c	Describe roles of funder(s), sponsor(s), and / or institution(s), if any, in developing the protocol	16
Rationale	#6	Describe the rationale for the review in the context of what is already known	3-5
Objectives	#7	Provide an explicit statement of the question(s) the review will address with reference to participants, interventions, comparators, and outcomes (PICO)	5 (5-8)
Eligibility criteria	#8	Specify the study characteristics (such as PICO, study design, setting, time frame) and report characteristics (such as years considered, language, publication status) to be used as criteria for eligibility for the review	5-13
Information sources	#9	Describe all intended information sources (such as electronic databases, contact with study authors, trial registers or other grey literature sources) with planned dates of coverage	8-9
Search strategy	#10	Present draft of search strategy to be used for at least one electronic database, including planned limits, such that it could be repeated	9-10
Study records - data management	#11a	Describe the mechanism(s) that will be used to manage records and data throughout the review	10
Study records - selection process	#11b	State the process that will be used for selecting studies (such as two independent reviewers) through each phase of the review (that is, screening, eligibility and inclusion in meta-analysis)	10
Study records - data collection process	#11c	Describe planned method of extracting data from reports (such as piloting forms, done independently, in duplicate), any processes for obtaining and confirming data from investigators	10
Data items	#12	List and define all variables for which data will be sought (such as PICO items, funding sources), any pre-planned data assumptions and simplifications	11

For peer review only

Outcomes and prioritization	#13	List and define all outcomes for which data will be sought, including prioritization of main and additional outcomes, with rationale	12-13
Risk of bias in individual studies	#14	Describe anticipated methods for assessing risk of bias of individual studies, including whether this will be done at the outcome or study level, or both; state how this information will be used in data synthesis	13
Data synthesis	#15a	Describe criteria under which study data will be quantitatively synthesised	7-8
	#15b	If data are appropriate for quantitative synthesis, describe planned summary measures, methods of handling data and methods of combining data from studies, including any planned exploration of consistency (such as I2, Kendall's τ)	N/A
	#15c	Describe any proposed additional analyses (such as sensitivity or subgroup analyses, meta-regression)	14
	#15d	If quantitative synthesis is not appropriate, describe the type of summary planned	14-15
Meta-bias(es)	#16	Specify any planned assessment of meta-bias(es) (such as publication bias across studies, selective reporting within studies)	15
Confidence in cumulative evidence	#17	Describe how the strength of the body of evidence will be assessed (such as GRADE)	15-16

The PRISMA-P checklist is distributed under the terms of the Creative Commons Attribution License CC-BY 4.0. This checklist can be completed online using https://www.goodreports.org/, a tool made by the EQUATOR Network in collaboration with Penelope.ai

For peer review only

BMJ Open

HOW CAN METRICS BE USED TO ROUTINELY MONITOR ANTIBIOTIC USAGE AND DEFINE (IN)APPROPRIATE ANTIBIOTIC USAGE IN SECONDARY CARE? A SYSTEMATIC REVIEW PROTOCOL

Journal:	*BMJ Open*
Manuscript ID	bmjopen-2018-026792.R1
Article Type:	Protocol
Date Submitted by the Author:	15-Feb-2019
Complete List of Authors:	Patel, Selina; University College London, Institute of Health Informatics<br>Jhass, Arnoupe; University College London, Department of Primary Care and Population Health, Institute of Epidemiology and Health Care<br>Hopkins, Susan; Public Health England<br>Shallcross, Laura; University College London, Institute of Health Informatics
**Primary Subject Heading**:	Public health
Secondary Subject Heading:	Infectious diseases, Research methods
Keywords:	antibiotic resistance, antibiotic usage, secondary care, high resource

SCHOLARONE™
Manuscripts

TITLE: HOW CAN METRICS BE USED TO ROUTINELY MONITOR ANTIBIOTIC USAGE AND DEFINE (IN)APPROPRIATE ANTIBIOTIC USAGE IN SECONDARY CARE? A SYSTEMATIC REVIEW PROTOCOL

CORRESPONDING AUTHOR: Selina Patel

Email: selina.patel.17@ucl.ac.uk

Address: 222 Euston Road, London, NW1 2DA

AUTHORS: Selina Patel[1], Arnoupe Jhass[2], Susan Hopkins[3], Laura Shallcross[1]

WORD COUNT: 3985

KEYWORDS: antibiotic resistance; antibiotic usage; secondary care; high-resource

ABSTRACT

Introduction

Ecological and individual-level evidence indicates that there is an association between level of antibiotic exposure and the emergence and spread of antibiotic resistance. The Global Point Prevalence Survey in 2015 estimated that 34.4% of hospital inpatients globally received at least one antimicrobial. Antimicrobial stewardship to optimise antibiotic usage in secondary care can reduce the high risk of patients acquiring and transmitting drug resistant infections in this setting. However, differences in the availability of data on antibiotic usage in this context make it difficult to develop a consensus of how to comparably monitor antibiotic prescribing patterns across secondary care. This hinders our ability to monitor progress in our attempts to mitigate antibiotic resistance.

[1] Public Health Data Science, Institute of Health Informatics, University College London
[2] Department of Primary Care & Population Health, Institute of Epidemiology and Health Care, University College London
[3] Public Health England

For peer review only

Methods and Analysis

We will search Medline (Ovid), Embase (Ovid), CINAHL, Cochrane Central Register of Controlled Trials and websites of key organisations for published reports where an attempt to measure antibiotic usage among adult in-patients in high-income hospital settings has been made. Two independent reviewers will screen the studies for eligibility, extract data and assess the study quality and risk of bias using the Newcastle-Ottawa scale. A description of the methods and measures used in antibiotic consumption surveillance will be presented. An adaptation of the APEASE (Affordability, Practicability, Effectiveness, Acceptability, Side-effects, Equity) framework will be used to consider the practicality of implementing different approaches to measuring antibiotic usage in secondary care settings. A descriptive comparison of definitions and estimates of (in)appropriate antibiotic usage will also be carried out.

Ethics and Dissemination

Ethical approval is not required for this study as no primary data will be collected. The results of this review will be published in relevant peer reviewed journals and presented at relevant conferences or meetings where possible. This review will inform future approaches to scale up antibiotic consumption surveillance strategies to attempt to maximise impact through standardisation.

PROSPERO Registration Number

CRD42018103375

ARTICLE SUMMARY

Strengths and limitations of this study

This study protocol follows the recommendations outlined in the Preferred Reporting Items for Systematic Review and Meta-analysis Protocols.

This protocol has been registered on the International Prospective Register of Systematic Reviews.

For peer review only

The selection of studies, data extraction and study quality assessments will be conducted by two independent reviewers.

The exclusion of studies which do not report the total number of patients may exclude very large scale antibiotic consumption surveillance projects.

The information available in some published studies may be too limited to assess surveillance methodologies using the APEASE framework.

For peer review only

Introduction

It is estimated that by the year 2050, 10 million deaths per year will be attributable to antimicrobial resistance (AMR).[1] There is good evidence from ecological studies that at the population level higher rates of antibiotic resistance are associated with high levels of antibiotic consumption.[2] There are increasing data to indicate that antibiotic exposure promotes resistance at the individual level also.[3] Nationally aggregated antibiotic consumption across 65 countries is estimated to range between 4.4 and 64.4 Defined Daily Doses per 1000 inhabitants per day.[4] The Global Point Prevalence Survey estimated that 34.4% of adult inpatients admitted to 335 hospitals globally received at least one antimicrobial, and that 8.4% of adult inpatients were treated with a systemic antibacterial for a healthcare associated infection(s).[5] Antimicrobial stewardship (AMS) to optimise the use of antimicrobials and safely reduce the total quantity of antimicrobials consumed is a recognised approach to control the emergence and spread of AMR. Surveillance of antimicrobial consumption underpins the ability to monitor the impact of AMS interventions and to measure variation in appropriate/inappropriate antibiotic prescribing over time. Despite this, conventional metrics of antibiotic usage which enable comparisons across settings in secondary care have not been established.[6] In the currently published literature, there is also a lack of definitions or quantitative estimates of inappropriate antibiotic consumption in secondary care.

Measuring antibiotic prescribing in secondary care

A range of strategies are being adopted to monitor and improve antibiotic prescribing in hospital. In Australia and England, surveillance systems have been established to measure antibiotic use in hospitals based on dispensing data from hospital pharmacies, converted into Defined Daily Doses.[7,8] A DDD is the assumed average maintenance dose per day for a drug for its main indication in adults.[9] DDDs are widely adopted to estimate quantities of antibiotic consumption and enable comparisons across drugs. However, the denominator of this measure in secondary care varies to make estimates less comparable, for example; 1000 occupied bed days, 1000 inhabitants, 1000 admissions.[10–13] This variation

For peer review only

is likely to reflect differences in the availability of data across high-income secondary care settings. These metrics do not consider patient case-mix, nor is there is not a widely accepted methodology to apply to estimates to account for this. Consequently, any comparisons drawn between quantities of antibiotic usage across healthcare settings must be cautiously interpreted.

It is recommended that antimicrobial consumption surveillance should combine aggregate data with patient-level data to better understand patterns of prescribing.[4] In secondary care, patient-level data on prescribing and prescribing associated factors are usually obtained through point prevalence surveys, representing a single point in time.[14] However, secondary care is a dynamic setting and several clinicians may be involved in different stages of the prescribing process including : antibiotic initiation, treatment selection, clinical review, de-escalation and cessation of treatment. This contrasts with primary care, where patients usually have a single consultation which may result in an antibiotic prescription. This makes understanding patterns of prescribing within hospitals more difficult to map and highlights the usefulness of using patient-level prescribing data linked to the prescriber, to monitor patterns of antimicrobial usage and stewardship as patients move through the hospital.

Measuring antibiotic stewardship

In the UK, the Commissioning for Quality and Innovation (CQUIN) has established performance management targets to financially incentivize reductions in antibiotic use in primary and secondary care. This includes targets to promote prescribing review within 72 hours. In most hospitals, adherence to this target is monitored by auditing a sample of prescriptions. [15,16]This approach is time consuming and cannot capture the range of factors that might have an important bearing on the quality of the review, such as patient characteristics or the seniority or specialty of individuals who were involved in the prescribing process. Such factors limit the ability to make inferences about the quality of AMS in different hospitals. Importantly existing measures provide little insight into where hospitals should target resources in their setting to have the greatest impact on antibiotic prescribing.

For peer review only

The National Antimicrobial Prescribing Survey (NAPS) measures similar antibiotic stewardship behaviours, however it goes further by estimating inappropriate prescribing. These data are collected through point prevalence surveys of all patients in the hospital where possible. Inappropriateness is defined in this survey as prescribing the wrong antibiotic spectrum, incorrect dose/frequency/duration/route, and prescribing when an antimicrobial is not needed. Decisions on inappropriateness are made by a member of the clinical team's assessment and are reported as prevalence of prescriptions. In 2017, 17,366 patients were submitted to NAPS with 26,661 prescriptions from 314 participating hospitals; 26,227 of these were assessed for inappropriateness.[17] This is a large data collection workload which, in Australia, is only a proportion of the NAPS, and runs annually alongside NAUSP and the Surgical NAPS. It is also a methodology which could be subject to variation between auditors when assessing inappropriateness of a broad range of prescriptions. Manual audits therefore seem labor-intensive and impractical for future attempts to scale up data collection of big patient-level datasets to understand more granular patterns of prescribing in secondary care over time.

Variations exist in the length of time between data collection and publication of results across surveillance programmes. The 2016 NAUSP data were published in 2018 while the 2017 SWEDRES|SVARM report on the consumption of antibiotics and antibiotic resistance in Sweden published data collected in 2017 that same year.[10,13] This likely reflects extensive differences in the availability of data, surveillance infrastructure and the digital maturity of healthcare systems which can be observed across high-income countries. The widespread establishment of electronic prescribing systems in the USA and the proposed investments in electronic prescribing systems in the UK and Australia, provides a potential opportunity to digitize systems to monitor antimicrobial usage and stewardship in secondary care, and capture patient-level prescribing data over time to understand patterns of prescribing.[18,19] This would enable descriptions of patterns of prescribing within hospitals to better understand the drivers of appropriate and inappropriate antibiotic usage in secondary care, however would

rely upon systems which facilitate good documentation of stewardship behaviours and prescriber decision making.

As high-income settings answer the WHO call to increase the availability of antimicrobial usage data, there is a need to reach a consensus on how to interrogate this data and be able to draw comparisons between hospital sites.[20]. Previous attempts to establish a conventional approach have failed to address all of the issues which we have already highlighted. Recently, two studies attempted to establish a consensus on quality and quantity indicators for measuring antibiotic use in secondary care using systematic review and RAND-modified Delphi methods.[21,22] However, neither study considered differences in the availability of data and resources between hospital settings, the practicability of implementing these indicators in surveillance strategies, or the strengths and limitations of the measures. To develop robust systems to monitor antibiotic usage in secondary care requires the development of simple measures which enable comparisons of antibiotic usage over time and across specialties. Critically, these measures must be co-developed with clinicians and pharmacists, and be based on data that are routinely available, since few hospitals have the resources to conduct regular audits of antimicrobial use.

The APEASE (Affordability, Practicability, Effectiveness and cost-effectiveness, Acceptability, Side-effects/safety, and Equity) criteria for designing interventions provides a framework within which researchers can comprehensively consider the context-specific suitability of an intervention.[23] It has been demonstrated repeatedly within the field in which it was developed to assess the suitability of behaviour change intervention functions.[24–27] However it is also provides the potential to be a useful tool when considering the complexities of identifying a suitable standardized antibiotic consumption surveillance methodology for the high-income secondary care setting.

This review will therefore study attempts to quantify total systemic antibiotic usage, appropriate antibiotic usage and inappropriate usage in high income settings in secondary care. This review will provide a summary of the ways in which antibiotic usage can be measured alongside a narrative about the

For peer review only - http://bmjopen.bmj.com/site/about/guidelines.xhtml

practicality of employing these metrics in secondary care based on an adaptation of the APEASE framework.

OBJECTIVES

The aim of this review is to provide an outline of the methodologies to quantify antibiotic usage among in-patients in secondary care. The objectives are:

- To identify the data sources and metrics which have most frequently been used to represent antibiotic usage in secondary care.
- To assess the suitability of antibiotic use surveillance methodologies for use in routine surveillance of antibiotic usage based on the APEASE criteria.
- To document the variation in definitions and estimates of appropriate and inappropriate antibiotic usage.

METHODS

The following methods are based on the framework provided by the 'Preferred Reporting Items for Systematic review and Meta-Analysis Protocols (PRISMA-P) checklist.[28] This review will begin in August 2018 and we aim to complete the review by September 2019.

Eligibility Criteria

Types of Studies

Study inclusion criteria:

- The article must make an attempt(s) to quantify antibiotic usage in at least 100 adult patients (>17 years old) among in-patients in secondary care. It is expected quantities of antibiotic use and patterns of prescribing in non-adult inpatients is likely to be significantly different to those in adults, so this review will focus on the adult inpatient, high-income secondary care setting.

For peer review only

- Articles which focus on antibiotic usage among individuals with specific conditions or specific demographic groups will be included.
- Those studies which do not provide enough information for a complete assessment using the APEASE criteria will be included in the final review, but excluded from this stage of analysis.
- The nature of these criteria is such that we would expect a range of study designs which are based on both observation and intervention, and which have attempted to estimate antibiotic usage in secondary care for a variety of purposes.

Study exclusion criteria:

- This review will not include trials of antibiotic therapies in secondary care or reviews of the literature.
- Articles which describe a methodology for measuring antibiotic usage and which do not attempt to implement it to estimate antibiotic usage will be excluded.
- Articles which are not written in English-language and are based on non-human participants will be excluded.
- If the number of adult in-patients included in the study is not stated, the article will be excluded from the review.

Types of Participants

The study must be based on dispensing, prescribing, survey/audit or sales data collected from the in-patient secondary care setting. It is expected that hospitals in high-income settings will be very different to those in low-resource settings in both the availability of data and resources available for stewardship, therefore this review will focus on hospitals in high-income countries as classified by the World Bank only (**Table S1**).[29] When the country is not classified in the World Bank lending groups, the article will be included if the gross national income (GNI) per capita is 12,056 USD or more. Articles must provide data which quantifies antibiotic usage among adult patients (>17-years old).

For peer review only

Types of Interventions

This review will consider all attempts to measure antibiotic usage among at least 100 patients in the hospital in-patient setting.

Types of Comparators

There are no comparison groups for this review as it is an exploration to identify the methodologies of how antibiotic usage has been measured in secondary care, and how this has been used to define and estimate appropriate and inappropriate antibiotic usage.

Types of Outcome measures

Articles which report a measure of antibiotic usage in hospital will be included in this review.

Primary Outcomes

- Data source used to represent antibiotic usage.
- Metrics which attempt to quantify antibiotic usage.
- An analysis of the suitability of existing metrics of antibiotic usage for use in routine practice to monitor antibiotic usage based on an adaptation of the APEASE framework (detailed in *Data Synthesis*).[23]

Secondary Outcomes

- Quantitative definitions of appropriate and inappropriate antibiotic usage.
- Comparison of the definitions of appropriate and inappropriate prescribing in secondary care.
- Description of variations in estimates of appropriate and inappropriate prescribing in secondary care.

Information Sources

For peer review only

Electronic Searches

The following databases will be searched for relevant articles:

- Medline (Ovid);
- Embase (Ovid);
- Cumulative Index to Nursing and Allied Health Literature (CINAHL);
- Cochrane Central Register of Controlled Trials;

The search strategy will be developed in Medline and then adapted appropriately for use in each database. The search will be conducted across all databases on 2nd August 2018.

Website Searches

In order to capture reports from the grey literature, the websites of relevant organisations will be searched (**Table S2**). The list of relevant organisations expands on a list originally conceived by *Benic et. al* based on discussion between SP, AJ and LS.[30] This review aims to consider the suitability of existing metrics of antibiotic usage in routine hospital surveillance of antibiotic usage; therefore it is essential that approaches to do this on a large scale such as through National Action Plans, which are often published in the grey literature, should be captured in our search. This search will be conducted in February 2019, the specific dates of which will be reported in the final review.

Search Strategy

The provisional search strategy is adapted from the work of *Benic et. al* based on four concepts: antibiotics; utilisation; measure; hospital (**Table 1**).[30] The search strategy will be established in MEDLINE and then adapted to the other 3 databases included in the search. No restrictions will be placed on the search to maximize sensitivity.

Table 1. Medline (Ovid) provisional search terms.

For peer review only - http://bmjopen.bmj.com/site/about/guidelines.xhtml

Search Concept	Search Terms
**Antibiotic**	1. Anti-Bacterial Agents/ad, dt, sd, tu, th, ut [Administration & Dosage, Drug Therapy, Supply & Distribution, Therapeutic Use, Therapy, Utilization]
	2. Antibiotic Prophylaxis/ec, mt, sn, td, ut [Economics, Methods, Statistics & Numerical Data, Trends, Utilization]
	3. (anti?biotic? or anti?microbial? or anti?bacterial?).ab,ti.
	4. 1 or 2 or 3
**Utilisation**	5. Drug Prescriptions/
	6. Drug Utilization/
	7. "Drug Utilization Review"/cl, ec, mt, st, sn, td, ut [Classification, Economics, Methods, Standards, Statistics & Numerical Data, Trends, Utilization]
	8. ((anti?biotic? or anti?microbial?) adj3 (prescri* or consumption or utili?ation or usage or "use" or dispens* or sale?)).ab,ti.
	9. 5 or 6 or 7 or 8
**Measurement**	10. BENCHMARKING/cl, ec, mt, st, sn, td, ut [Classification, Economics, Methods, Standards, Statistics & Numerical Data, Trends, Utilization]
	11. (intervention adj5 (prescri* or stewardship or "use" or utili?ation or usage or consumption)).ab,ti
	12. ((anti?biotic? or anti?microbial?) adj4 (estimat* or quanitf* or metric? or monitor* or surveillance or prevalence or survey or audit)).ab,ti.
	13. (electronic prescri* or e?prescri*).ab,ti.
	14. 10 or 11 or 12 or 13
**Secondary Care**	15. Secondary Care/
	16. Hospitals/
	17. hospital*.ab,ti.

For peer review only

	18. 15 or 16 or 17
	19. 4 and 9 and 14 and 18
**Legend.** .**ab**, abstract; **ti**., title; **adj3**, indicates two words next to each other in any order with up to 2 words in between; /, indicates a Medical Subject Heading (MeSH); *****, denotes any truncation; **?**, denotes one character or no character. All other abbreviations are elaborated upon in [ ] brackets in the table.	

For peer review only

Study Records

Data Management

The articles will be uploaded from the relevant database to *Mendeley* reference manager software. The reports will be exported to the web-based, *EPPI Reviewer 4* systematic review software which will be used to remove duplicate articles. Each stage of the screening process will be carried out in *EPPI Reviewer 4* using a standardised form of article eligibility. The online form for article eligibility will be piloted before the start of the screening process and amended as required to ensure it is fit for purpose. The data will be extracted and entered into a standardised, piloted, pre-created online data extraction form in *EPPI Reviewer 4* in duplicate by two independent reviewers (SP and AJ).

Selection Process

The unique articles captured by the search will undergo a title pre-screening by a single reviewer (SP). The remaining articles will then be screened based on title and abstract by two independent reviewers in duplicate (SP and AJ) ; when study eligibility is not mutually agreed upon or is ambiguous from the title and abstract only, the article will be admitted into the full-text review stage. The selected articles will undergo full-text review by two independent reviewers in duplicate (SP and AJ). Any disagreements between authors at any stage will be resolved through a third reviewer (LS). The reasons

for the exclusion of these studies from this review will be documented for reference and the study selection process will be presented as a PRISMA flow diagram.[28]

Data Collection Process

The data will be collected in duplicate by two independent reviewers (SP and AJ) from articles selected for inclusion in the review. The data will be entered into pre-created, piloted forms on EPPI Reviewer 4: systematic review software. Any disagreement will be resolved through consultation with a third author (LS).

Data Items

We will extract data on:

- **Study** (study design, country, population, service setting such as size of hospital, objective).
- **Data type used to describe antibiotic usage** (data source, method of collection, frequency of the data collection, reason for data collection e.g. national surveillance or research, who collected the data, level of detail in the data and data type).
- **Metrics used to measure antibiotic usage** (numerator, denominator, units (such as per admission), level at which the metric is presented (such as by specialty).
- **Criteria used to define appropriate and inappropriate antibiotic usage**.
- **Data type used to quantify appropriate and inappropriate antibiotic usage** (data source, method of collection, level of detail in the data and data type).
- **Quantitative estimates of appropriate and inappropriate antibiotic usage** (numerator, denominator, units (such as per admission), level at which the metric is presented (such as by specialty) and what the metric is currently used for (such as national or hospital-level surveillance)).

For peer review only

- **Study quality and selection bias of those studies which provide an estimate of appropriate and inappropriate antibiotic usage** (outlined below under *Assessment of Risk of Bias*).

Outcomes Prioritisation

The main outcome of this review is a description of the data sources and metrics which have been used to measure antibiotic usage and to define appropriate and inappropriate antibiotic usage in hospitals. This will include a description of the teams most frequently involved in this type of work in order to identify those groups who are often facilitators of this surveillance.

This will then be used to consider each surveillance method within an adaptation of the APEASE framework to assess the practicality of each method and metric for use in routine surveillance of antibiotic usage; this method is detailed in '*Data synthesis*'.[23] This aims to provide detail to inform the methodology of sustainable antibiotic usage surveillance programmes. We will also document if it is stated that the metric is currently being routinely used for any purpose such as national level surveillance. This will identify reasons why antibiotic usage surveillance is already being implemented, highlight motivations for the allocation of resources to antimicrobial consumption surveillance and combined with methodological details will reveal programmes where existing infrastructure may be extended for use to routinely monitor antibiotic usage within hospitals. If the metric has been used to define and estimate (in)appropriate antibiotic usage, this will be used to explore the variation in the definitions and estimates of inappropriate antibiotic usage in hospitals. It is anticipated that such estimations will have been made using a multitude of methodologies and measures. The extent to which quantitative estimates of inappropriate prescribing can be directly compared may therefore be limited.

Assessment of Risk of Bias in Individual Studies

All metrics of antibiotic consumption included in the final review must have been utilized in the secondary care setting to capture the antibiotic usage data for at least 100 patients. This is to ensure some

level of demonstration of the feasibility of implementing the measures. For these descriptive purposes, it is not appropriate to use a risk of bias framework.

For studies included in the full-text review stage and which attempt to make a quantitative estimate of appropriate or inappropriate antibiotic usage in the secondary care setting, the Newcastle-Ottawa scale to assess risk of bias will be used to determine the inclusion of the estimate in the review.[31]

Data synthesis

The data will undergo a descriptive synthesis to provide insight into the methodologies to implement antibiotic usage metrics in secondary care. We will record the frequency of use of different data sources by level (individual to national) and by source (such as electronic prescribing or audits). This will examine the type of data which is currently being used to monitor antibiotic usage and consequently the level detail in which we are able to examine prescribing currently. We will also identify the frequency of use of each metric to measure: volume, type, duration, stewardship activity and prevalence/incidence of antibiotic use. As well as identify the level at which these metrics are most frequently presented such as by specialty or nationally. The teams involved in these studies to collect and analyse the data will also be recorded.

An adaptation of the APEASE framework will be used to assess the feasibility of metrics for use in routine monitoring of antibiotic usage. The APEASE framework was designed to aid the context-specific design and selection of interventions to promote health behaviour change.[23] The framework acronym is elaborated to: a – affordability; p – practicability; e – effectiveness and cost-effectiveness; a – acceptability; s – side-effects/safety; e – equity. This original framework will be adapted to inform the establishment of a consensus to monitor antibiotic usage in the high-income secondary care setting [**Table 2**].

Table 2. An adaptation of the APEASE framework to assess the feasibility of metrics for use in routine surveillance.

For peer review only - http://bmjopen.bmj.com/site/about/guidelines.xhtml

**A**	[Affordability] Resources required to sustainably implement the metric. For example, person time to audit / new system roll-out.
**P**	[Practicability] Can the metric be delivered? Is there sustainable access to data and resources?
**E**	[Effectiveness] Does the surveillance method capture sufficient data?
**A**	[Acceptability] Does it represent relevant data to monitor antibiotic usage and appropriateness of antibiotic usage?
**S**	[Side-effects] Bias of metrics
**E**	[Equity] Is it feasible for hospital trusts of varying level of resources to all implement the surveillance methodology?

This analysis can then be used to inform recommendations based on the available literature regarding a sustainable, conventional strategy to monitor antibiotic usage and the resources which would be required to practically implement such a surveillance strategy.

The variation between comparable hospital-level estimates of (in)appropriate antibiotic usage will be represented using a funnel plot of inappropriate prescribing estimates against patient numbers and 95% confidence intervals.

Meta-bias(es)

The systematic review will be conducted in line with this protocol and any aspects of the method which deviate from the original protocol will be reported in the final review. Only articles written in English-language will be included in this review; this is due to the language spoken by the authors.

Confidence in Cumulative Evidence

This is a descriptive review to consider the methodologies which have been used to measure antibiotic usage and to define appropriate and inappropriate antibiotic prescribing in hospitals. The methodologies

For peer review only

will be considered in terms of the APEASE criteria and it is therefore not necessary to consider the quality of quantitative evidence for all studies. Observational studies which attempt to make a quantitative estimate of appropriate or inappropriate antibiotic usage in the secondary care setting, the Newcastle-Ottawa scale to assess risk of bias will be used to determine the inclusion of the estimate in the review.[31]

Patient and Public Involvement

Patients and members of the public were not involved in the development of this protocol.

Contributors

The study was conceived by SP, SH and LS. SP developed the eligibility criteria, search strategy, risk of bias assessment and the data extraction plans with guidance from LS. SP, AJ and LS developed the website search list. All authors contributed to and reviewed the final manuscript.

Acknowledgements

The authors acknowledge the valuable technical guidance offered by Suvi Härmälä in the construction of the protocol.

Funding

This review was funded by the Economic and Social Research Council (ESRC); grant number: ES/P000592/1 awarded to SP. The ESRC was not involved in the development of this protocol.

Patient Consent

Not required.

Ethics Approval

Ethical approval is not required for this study.

For peer review only

CONFLICTS OF INTEREST

The authors declare no conflicts of interest.

BIBLIOGRAPHY

1. O'Neill, J. *TACKLING DRUG-RESISTANT INFECTIONS GLOBALLY: FINAL REPORT AND RECOMMENDATIONS THE REVIEW ON ANTIMICROBIAL RESISTANCE*. (2016).

2. Goossens, H., Ferech, M., Vander Stichele, R. & Elseviers, M. Outpatient antibiotic use in Europe and association with resistance: a cross-national database study. *Lancet* **365,** 579–587 (2005).

3. Costelloe, C., Metcalfe, C., Lovering, A., Mant, D. & Hay, A. D. Effect of antibiotic prescribing in primary care on antimicrobial resistance in individual patients: systematic review and meta-analysis. *BMJ* **340,** c2096 (2010).

4. World Health Organization. *WHO Report on Surveillance of Antibiotic Consumption*. (2018).

5. A., V. *et al.* Antimicrobial consumption and resistance in adult hospital inpatients in 53 countries: results of an internet-based global point prevalence survey. *Lancet Glob. Heal.* **6,** e619–e629 (2018).

6. Zanichelli, V. *et al.* Variation in antibiotic use among and within different settings: a systematic review. *J. Antimicrob. Chemother.* **73,** vi17-vi29 (2018).

7. Public Health England. English surveillance programme for antimicrobial utilisation and resistance (ESPAUR) report - GOV.UK. Available at: https://www.gov.uk/government/publications/english-surveillance-programme-antimicrobial-utilisation-and-resistance-espaur-report. (Accessed: 19th November 2018)

8. Australian Commission on Safety and Quality in Health Care. *Antimicrobial Use in Australian Hospitals 2016 annual report of the National Antimicrobial Utilisation Surveillance Program*.

For peer review only

(2018).

9. WHOCC. WHOCC - Definition and general considerations. Available at: https://www.whocc.no/ddd/definition_and_general_considera/. (Accessed: 24th January 2019)

10. SA Health. *Antimicrobial Use in Australian Hospitals: 2016 annual report of the National Antimicrobial Utilisation Surveillance Program Published by the Australian Commission on Safety and Quality in Health Care*. (2018).

11. Public Health England. *English Surveillance Programme for Antimicrobial Utilisation and Resistance (ESPAUR) 2017*. (2017).

12. Statens Serum Institute, National Veterinary Institute- Technical University of, Denmark & National Food Institute - Technical University of Denmark. *DANMAP 2017-Use of antimicrobial agents and occurrence of antimicrobial resistance in bacteria from food animals, food and humans in Denmark*.

13. Public Health Agency of Sweden & National Veterinary Institute. SWEDRES |SVARM 2017 2 A report on Swedish Antibiotic Utilisation and Resistance in Human Medicine (Swedres) and Swedish Veterinary Antibiotic Resistance Monitoring (Svarm) Editors.

14. ECDC. About ECDC Point prevalence survey. Available at: https://ecdc.europa.eu/en/healthcare-associated-infections-acute-care-hospitals/facts/about. (Accessed: 31st January 2019)

15. NHS England. NHS England » Commissioning for Quality and Innovation (CQUIN) Guidance for 2017-2019. Available at: https://www.england.nhs.uk/publication/commissioning-for-quality-and-innovation-cquin-guidance-for-2017-2019/. (Accessed: 24th August 2018)

16. NHS England. NHS England » 2017/19 CQUIN. Available at: https://www.england.nhs.uk/nhs-standard-contract/cquin/cquin-17-19/. (Accessed: 13th December 2018)

For peer review only

17. Australian Commission on Safety and Quality in Health Care. *Antimicrobial prescribing practice in Australian hospitals*. (2018).

18. Drug errors cause appalling harm and deaths, says Hunt - BBC News. Available at: https://www.bbc.co.uk/news/health-43161929. (Accessed: 14th August 2018)

19. Australian Government Department of Health. Improving Access to Medicines – ePrescribing for safer medicines. Available at: http://www.health.gov.au/internet/budget/publishing.nsf/Content/budget2018-factsheet14.htm. (Accessed: 24th August 2018)

20. World Health Organization. *Global Action Plan on Antimicrobial Resistance*. (2015).

21. Monnier, A. A. *et al.* Quality indicators for responsible antibiotic use in the inpatient setting: a systematic review followed by an international multidisciplinary consensus procedure. *J. Antimicrob. Chemother.* **73,** vi30-vi39 (2018).

22. Stanić Benić, M. *et al.* Metrics for quantifying antibiotic use in the hospital setting: results from a systematic review and international multidisciplinary consensus procedure. *J. Antimicrob. Chemother.* **73,** vi50-vi58 (2018).

23. Michie, S., Atkins, L. & West, R. *The Behaviour Change Wheel Book - A Guide To Designing Interventions*. (2014).

24. Suntornsut, P. *et al.* Barriers and Recommended Interventions to Prevent Melioidosis in Northeast Thailand: A Focus Group Study Using the Behaviour Change Wheel. *PLoS Negl. Trop. Dis.* **10,** e0004823 (2016).

25. Sinnott, C. *et al.* Improving medication management in multimorbidity: development of the MultimorbiditY COllaborative Medication Review And DEcision Making (MY COMRADE)

For peer review only

intervention using the Behaviour Change Wheel. *Implement. Sci.* **10,** 132 (2015).

26. Cadogan, C. A. *et al.* Development of an intervention to improve appropriate polypharmacy in older people in primary care using a theory-based method. *BMC Health Serv. Res.* **16,** 661 (2016).

27. Mc Sharry, J., Murphy, P. J. & Byrne, M. Implementing international sexual counselling guidelines in hospital cardiac rehabilitation: development of the CHARMS intervention using the Behaviour Change Wheel. *Implement. Sci.* **11,** 134 (2016).

28. Shamseer, L. *et al.* Preferred reporting items for systematic review and meta-analysis protocols (PRISMA-P) 2015: elaboration and explanation. *BMJ* **350,** g7647 (2015).

29. Group, T. W. B. World Bank Country and Lending Groups. Available at: https://datahelpdesk.worldbank.org/knowledgebase/articles/906519-world-bank-country-and-lending-groups.

30. Stanic Benic, M. *et al.* Metrics for quantifying antibiotic use in the hospital setting: results from a systematic review and international multidisciplinary consensus procedure. *J. Antimicrob. Chemother.* **73,** vi50-vi58 (2018).

31. Wells, G. *et al.* The Newcastle-Ottawa Scale (NOS) for assessing the quality of nonrandomised studies in meta-analyses. Available at: http://www.ohri.ca/programs/clinical_epidemiology/oxford.asp. (Accessed: 31st July 2018)

For peer review only

SUPPLEMENTARY MATERIALS

Table S1. ***World Bank Country Lending Groups Classification: High-Income Economies ($12,056 GNI per Capita or more)***

Accessed on: 23.08.18. Available at: https://datahelpdesk.worldbank.org/knowledgebase/articles/906519-world-bank-country-and-lending-groups

Countries				
Andorra	Germany	Oman	Antigua and Barbuda	Gibraltar
Palau	Argentina	Greece	Panama	Aruba
Greenland	Poland	Australia	Guam	Portugal
Austria	Hong Kong SAR, China	Puerto Rico	The Bahamas	Hungary
Qatar	Bahrain	Iceland	San Morino	Barbados
Ireland	Saudi Arabia	Belgium	Isle of Man	Seychelles
Bermuda	Israel	Singapore	British Virgin Islands	Italy
Sint Maarten (Dutch)	Brunei Darussalam	Japan	Slovak Republic	Canada
Korea Rep.	Slovenia	Cayman Islands	Kuwait	Spain
Channel Islands	Latvia	St. Kitts and Nevis	Chile	Liechtenstein
St. Martin (French)	Croatia	Lithuania	Sweden	Curaçao
Luxembourg	Switzerland	Cyprus	Macao SAR, China	Taiwan, China
Czech Republic	Malta	Trinidad and Tobago	Denmark	Monaco
Turks and Caicos Islands	Estonia	Netherlands	United Arab Emirates	Faroe Islands
New Caledonia	United Kingdom	Finland	New Zealand	United States
France	Northern Mariana Islands	Uruguay	French Polynesia	Norway
Virgin Islands (U.S.)				

For peer review only

Table S2. ***Website Search List***

Expanded upon from:

Stanic Benic, M. *et al.* Metrics for quantifying antibiotic use in the hospital setting: results from a systematic review and international multidisciplinary consensus procedure. *J. Antimicrob. Chemother.* **73,** vi50-vi58 (2018).

	**Organisation**	**Website(s)**
1	Africa CDC: African Union	https://au.int/en/africacdc
2	Agency for Healthcare Research and Quality	www.ahrq.gov/
3	Antibiotic Action	http://antibiotic-action.com/
4	Alliance for the Prudent Use of Antibiotics (APUA)	www.apua.org/
5	Australian Commission on Safety and Quality in Health Care | Antimicrobial Use and Resistance in Australia (AURA)	https://www.safetyandquality.gov.au/antimicrobial-use-and-resistance-in-australia/
6	Australian Government Department of Health	www.health.gov.au/
7	British Society for Antimicrobial Chemotherapy	http://www.bsac.org.uk/
8	British Infection Association	https://www.britishinfection.org/
9	Center for Disease Dynamics, Economics & Policy (CDDEP)	https://www.cddep.org/ https://resistancemap.cddep.org/AntibioticUse.php
10	Canadian Foundation for Healthcare Improvement	https://www.cfhi-fcass.ca/
11	Centers for Disease Control and Prevention (CDC)	https://www.cdc.gov/
12	Care Quality Commission	https://www.cqc.org.uk/
13	European Centre for Disease Control and Prevention (ECDC)	https://ecdc.europa.eu/en/home
14	European Society of Clinical Microbiology and Infectious Diseases (ESCMID)	https://www.escmid.org/
15	Healthcare Infection Association	https://www.britishinfection.org/
18	Institute for Healthcare Improvement: Australian Council for Safety and Quality Health Care	http://www.ihi.org/resources/Pages/OtherWebsites/AustralianCouncilforSafetyandQualityinHealthCare.aspx
19	International Society of Chemotherapy Infection and Cancer (ISC)	www.ischemo.org/
20	International Society of Infectious Diseases	www.isid.org/
21	National Institute for Health and Care Excellence (NICE)	https://www.nice.org.uk/
22	National Quality Measures Clearinghouse	https://www.qualitymeasures.ahrq.gov/
23	NHS Improvement	https://improvement.nhs.uk/
24	Public Health Agency of Canada	www.publichealth.gc.ca/

For peer review only

25	Public Health England	https://www.gov.uk/government/organisations/public-health-england<br>https://fingertips.phe.org.uk/profile/amr-local-indicators
26	Public Health Surveillance: New Zealand Ministry of Health & Institute of Environmental Science Research Ltd (ESR)	https://surv.esr.cri.nz/index.php
27	RAND Corporation	www.rand.org/
28	ReAct group	www.reactgroup.org/
29	Swedish Strategic Programme against Antibiotic Resistance (STRAMA)	www.strama.se
30	Transatlantic Task Force on Antimicrobial Resistance (TATFAR)	http://www.cdc.gov/drugresistance/tatfar/
31	US Food and Drug Administration	www.fda.gov/
32	World Health Organization	www.who.int/<br>http://www.afro.who.int/<br>https://www.paho.org/hq/<br>http://www.searo.who.int/en/<br>http://www.euro.who.int/en/home<br>http://www.emro.who.int/index.html<br>http://www.wpro.who.int/en/
33	Scottish Antimicrobial Prescribing Group	http://www.isdscotland.org/Health-Topics/Prescribing-and-Medicines/SAPG/

For peer review only

Reporting checklist for protocol of a systematic review.

Based on the PRISMA-P guidelines.

Instructions to authors

Complete this checklist by entering the page numbers from your manuscript where readers will find each of the items listed below.

Your article may not currently address all the items on the checklist. Please modify your text to include the missing information. If you are certain that an item does not apply, please write "n/a" and provide a short explanation.

Upload your completed checklist as an extra file when you submit to a journal.

In your methods section, say that you used the PRISMA-P reporting guidelines, and cite them as:

Moher D, Shamseer L, Clarke M, Ghersi D, Liberati A, Petticrew M, Shekelle P, Stewart LA. Preferred Reporting Items for Systematic Review and Meta-Analysis Protocols (PRISMA-P) 2015 statement. Syst Rev. 2015;4(1):1.

		Reporting Item	Page Number
Identification	#1a	Identify the report as a protocol of a systematic review	1
Update	#1b	If the protocol is for an update of a previous systematic review, identify as such	N/A

For peer review only

	#2	If registered, provide the name of the registry (such as PROSPERO) and registration number	2
Contact	#3a	Provide name, institutional affiliation, e-mail address of all protocol authors; provide physical mailing address of corresponding author	1
Contribution	#3b	Describe contributions of protocol authors and identify the guarantor of the review	1,18
	#4	If the protocol represents an amendment of a previously completed or published protocol, identify as such and list changes; otherwise, **state plan for documenting important protocol amendments**	17
Sources	#5a	Indicate sources of financial or other support for the review	18
Sponsor	#5b	Provide name for the review funder and / or sponsor	18
Role of sponsor or funder	#5c	Describe roles of funder(s), sponsor(s), and / or institution(s), if any, in developing the protocol	18
Rationale	#6	Describe the rationale for the review in the context of what is already known	4-7
Objectives	#7	Provide an explicit statement of the question(s) the review will address with reference to participants, interventions, comparators, and outcomes (PICO)	8+
Eligibility criteria	#8	Specify the study characteristics (such as PICO, study design, setting, time frame) and report characteristics (such	8-9

For peer review only

		as years considered, language, publication status) to be used as criteria for eligibility for the review	
Information sources	#9	Describe all intended information sources (such as electronic databases, contact with study authors, trial registers or other grey literature sources) with planned dates of coverage	10-11
Search strategy	#10	Present draft of search strategy to be used for at least one electronic database, including planned limits, such that it could be repeated	11-12
Study records - data management	#11a	Describe the mechanism(s) that will be used to manage records and data throughout the review	13
Study records - selection process	#11b	State the process that will be used for selecting studies (such as two independent reviewers) through each phase of the review (that is, screening, eligibility and inclusion in meta-analysis)	13
Study records - data collection process	#11c	Describe planned method of extracting data from reports (such as piloting forms, done independently, in duplicate), any processes for obtaining and confirming data from investigators	14
Data items	#12	List and define all variables for which data will be sought (such as PICO items, funding sources), any pre-planned data assumptions and simplifications	14

For peer review only

Outcomes and prioritization	#13	List and define all outcomes for which data will be sought, including prioritization of main and additional outcomes, with rationale	15
Risk of bias in individual studies	#14	Describe anticipated methods for assessing risk of bias of individual studies, including whether this will be done at the outcome or study level, or both; state how this information will be used in data synthesis	15-16
Data synthesis	#15a	Describe criteria under which study data will be quantitatively synthesised	16
	#15b	If data are appropriate for quantitative synthesis, describe planned summary measures, methods of handling data and methods of combining data from studies, including any planned exploration of consistency (such as I2, Kendall's τ)	17
	#15c	Describe any proposed additional analyses (such as sensitivity or subgroup analyses, meta-regression)	N/a
	#15d	If quantitative synthesis is not appropriate, describe the type of summary planned	16-17
Meta-bias(es)	#16	Specify any planned assessment of meta-bias(es) (such as publication bias across studies, selective reporting within studies)	17
Confidence in cumulative evidence	#17	Describe how the strength of the body of evidence will be assessed (such as GRADE)	17-18

For peer review only

The PRISMA-P checklist is distributed under the terms of the Creative Commons Attribution License CC-BY 4.0. This checklist can be completed online using https://www.goodreports.org/, a tool made by the EQUATOR Network in collaboration with Penelope.ai

For peer review only

BMJ Open

HOW CAN METRICS BE USED TO ROUTINELY MONITOR ANTIBIOTIC USAGE AND DEFINE (IN)APPROPRIATE ANTIBIOTIC USAGE IN SECONDARY CARE? A SYSTEMATIC REVIEW PROTOCOL

Journal:	*BMJ Open*
Manuscript ID	bmjopen-2018-026792.R2
Article Type:	Protocol
Date Submitted by the Author:	09-Apr-2019
Complete List of Authors:	Patel, Selina; University College London, Institute of Health Informatics<br>Jhass, Arnoupe; University College London, Department of Primary Care and Population Health, Institute of Epidemiology and Health Care<br>Hopkins, Susan; Public Health England<br>Shallcross, Laura; University College London, Institute of Health Informatics
**Primary Subject Heading**:	Public health
Secondary Subject Heading:	Infectious diseases, Research methods
Keywords:	antibiotic resistance, secondary care, high resource, antibiotic use

SCHOLARONE™
Manuscripts

TITLE: HOW CAN METRICS BE USED TO ROUTINELY MONITOR ANTIBIOTIC USAGE AND DEFINE (IN)APPROPRIATE ANTIBIOTIC USAGE IN SECONDARY CARE? A SYSTEMATIC REVIEW PROTOCOL

CORRESPONDING AUTHOR: Selina Patel

Email: selina.patel.17@ucl.ac.uk

Address: 222 Euston Road, London, NW1 2DA

AUTHORS: Selina Patel[1], Arnoupe Jhass[2], Susan Hopkins[3], Laura Shallcross[1]

WORD COUNT: 3961

KEYWORDS: antibiotic resistance; antibiotic usage; secondary care; high-resource

ABSTRACT

Introduction

Ecological and individual-level evidence indicates that there is an association between level of antibiotic exposure and the emergence and spread of antibiotic resistance. The Global Point Prevalence Survey in 2015 estimated that 34.4% of hospital inpatients globally received at least one antimicrobial. Antimicrobial stewardship to optimise antibiotic use in secondary care can reduce the high risk of patients acquiring and transmitting drug resistant infections in this setting. However, differences in the availability of data on antibiotic use in this context make it difficult to develop a consensus of how to comparably monitor antibiotic prescribing patterns across secondary care. This review will aim to document and critically evaluate methods and measures to monitor antibiotic use in secondary care.

[1] Public Health Data Science, Institute of Health Informatics, University College London
[2] Department of Primary Care & Population Health, Institute of Epidemiology and Health Care, University College London
[3] Public Health England

For peer review only

Methods and Analysis

We will search Medline (Ovid), Embase (Ovid), CINAHL, Cochrane Central Register of Controlled Trials and websites of key organisations for published reports where an attempt to measure antibiotic usage among adult in-patients in high-income hospital settings has been made. Two independent reviewers will screen the studies for eligibility, extract data and assess the study quality using the Newcastle-Ottawa scale. A description of the methods and measures used in antibiotic consumption surveillance will be presented. An adaptation of the APEASE (Affordability, Practicability, Effectiveness, Acceptability, Side-effects, Equity) framework will be used to consider the practicality of implementing different approaches to measuring antibiotic usage in secondary care settings. A descriptive comparison of definitions and estimates of (in)appropriate antibiotic usage will also be carried out.

Ethics and Dissemination

Ethical approval is not required for this study as no primary data will be collected. The results will be published in relevant peer reviewed journals and presented at relevant conferences or meetings where possible. This review will inform future approaches to scale up antibiotic consumption surveillance strategies to attempt to maximise impact through standardisation.

PROSPERO Registration Number

CRD42018103375

ARTICLE SUMMARY

Strengths and limitations of this study

This study protocol follows the recommendations outlined in the Preferred Reporting Items for Systematic Review and Meta-analysis Protocols.

This protocol has been registered on the International Prospective Register of Systematic Reviews.

For peer review only

The selection of studies, data extraction and study quality assessments will be conducted by two independent reviewers.

The exclusion of studies which do not report the total number of patients may exclude very large scale antibiotic consumption surveillance projects.

The information available in some published studies may be too limited to assess surveillance methodologies using the APEASE framework.

For peer review only

Introduction

It is estimated that by the year 2050, 10 million deaths per year will be attributable to antimicrobial resistance (AMR).[1] There is good evidence from ecological studies that at the population level higher rates of antibiotic resistance are associated with high levels of antibiotic consumption.[2] There are increasing data to indicate that antibiotic exposure promotes resistance at the individual level also.[3] Nationally aggregated antibiotic consumption across 65 countries is estimated to range between 4.4 and 64.4 Defined Daily Doses per 1000 inhabitants per day.[4] The Global Point Prevalence Survey estimated that 34.4% of adult inpatients admitted to 335 hospitals globally received at least one antimicrobial, and that 8.4% of adult inpatients were treated with a systemic antibacterial for a healthcare associated infection(s).[5] Antimicrobial stewardship (AMS) to optimise the use of antimicrobials and safely reduce the total quantity of antimicrobials consumed is a recognised approach to control the emergence and spread of AMR. Surveillance of antimicrobial consumption underpins the ability to monitor the impact of AMS interventions and to measure variation in appropriate/inappropriate antibiotic prescribing over time. Despite this, conventional metrics of antibiotic usage which enable comparisons across settings in secondary care have not been established.[6] In the currently published literature, there is also a lack of definitions or quantitative estimates of inappropriate antibiotic consumption in secondary care.

Measuring antibiotic prescribing in secondary care

A range of strategies are being adopted to monitor and improve antibiotic prescribing in hospital. In Australia and England, surveillance systems have been established to measure antibiotic use in hospitals based on dispensing data from hospital pharmacies, converted into Defined Daily Doses (DDD).[7,8] A DDD is the assumed average maintenance dose per day for a drug for its main indication in adults.[9] DDDs are widely adopted to estimate quantities of antibiotic consumption and enable comparisons across drugs. However, the denominator of this measure in secondary care varies to make estimates less comparable, for example; 1000 occupied bed days, 1000 inhabitants, 1000 admissions.[10–13]

For peer review only - http://bmjopen.bmj.com/site/about/guidelines.xhtml

This variation is likely to reflect differences in the availability of data across high-income secondary care settings. These metrics do not consider patient case-mix, nor is there is not a widely accepted methodology to apply to estimates to account for this. Consequently, any comparisons drawn between quantities of antibiotic usage across healthcare settings must be cautiously interpreted.

It is recommended that antimicrobial consumption surveillance should combine aggregate data with patient-level data to better understand patterns of prescribing.[4] In secondary care, patient-level data on prescribing and prescribing associated factors are usually obtained through point prevalence surveys, representing a single point in time.[14] However, secondary care is a dynamic setting and several clinicians may be involved in different stages of the prescribing process including: antibiotic initiation, treatment selection, clinical review, de-escalation and cessation of treatment. This contrasts with primary care, where patients usually have a single consultation which may result in an antibiotic prescription. This makes understanding patterns of prescribing within hospitals more difficult to map and highlights the usefulness of using patient-level prescribing data linked to the prescriber, to monitor patterns of antimicrobial usage and stewardship as patients move through the hospital.

Measuring antibiotic stewardship

In the UK, the Commissioning for Quality and Innovation (CQUIN) has established performance management targets to financially incentivize reductions in antibiotic use in primary and secondary care. This includes targets to promote prescribing review within 72 hours. In most hospitals, adherence to this target is monitored by auditing a sample of prescriptions.[15,16] This approach is time consuming and cannot capture the range of factors that might have an important bearing on the quality of the review, such as patient characteristics or the seniority or specialty of individuals who were involved in the prescribing process. Such factors limit the ability to make inferences about the quality of AMS in different hospitals. Importantly existing measures provide little insight into where hospitals should target resources in their setting to have the greatest impact on antibiotic prescribing.

For peer review only

The National Antimicrobial Prescribing Survey (NAPS) measures similar antibiotic stewardship behaviours, however it goes further by estimating inappropriate prescribing. These data are collected through point prevalence surveys of all patients in the hospital where possible. Inappropriateness is defined in this survey as prescribing the wrong antibiotic spectrum, incorrect dose/frequency/duration/route, and prescribing when an antimicrobial is not needed. Decisions on inappropriateness are made by a member of the clinical team's assessment and are reported as prevalence of prescriptions. In 2017, 17,366 patients were submitted to NAPS with 26,661 prescriptions from 314 participating hospitals; 26,227 of these were assessed for inappropriateness.[17] This is a large data collection workload which, in Australia, is only a proportion of the NAPS, and runs annually alongside NAUSP and the Surgical NAPS. It is also a methodology which could be subject to variation between auditors when assessing inappropriateness of a broad range of prescriptions. Manual audits therefore seem labor-intensive and impractical for future attempts to scale up data collection of big patient-level datasets to understand more granular patterns of prescribing in secondary care over time.

Variations exist in the length of time between data collection and publication of results across surveillance programmes. The 2016 NAUSP data were published in 2018 while the 2017 SWEDRES|SVARM report on the consumption of antibiotics and antibiotic resistance in Sweden published data collected in 2017 that same year.[10,13] This likely reflects extensive differences in the availability of data, surveillance infrastructure and the digital maturity of healthcare systems which can be observed across high-income countries. The widespread establishment of electronic prescribing systems in the USA and the proposed investments in electronic prescribing systems in the UK and Australia, provides a potential opportunity to digitize systems to monitor antimicrobial usage and stewardship in secondary care, and capture patient-level prescribing data over time to understand patterns of prescribing.[18,19] This could enable descriptions of patterns of prescribing within hospitals to better document appropriate and inappropriate antibiotic usage in secondary care, however it would rely upon systems which facilitate good documentation of stewardship behaviours and prescriber decision making.

For peer review only

As high-income settings answer the WHO call to increase the availability of antimicrobial use data, there is a need to reach a consensus on how to interrogate this data and be able to draw comparisons between hospital sites.[20]. Previous attempts to establish a conventional approach have failed to address all of the issues which we have already highlighted. Recently, two studies attempted to establish a consensus on quality and quantity indicators for measuring antibiotic use in secondary care using systematic review and RAND-modified Delphi methods.[21,22] However, neither study considered differences in the availability of data and resources between hospital settings, the practicability of implementing these indicators in surveillance strategies, or the strengths and limitations of the measures. To develop robust systems to monitor antibiotic usage in secondary care requires the development of simple measures which enable comparisons of antibiotic usage over time and across specialties. Critically, these measures must be co-developed with clinicians and pharmacists, and be based on data that are routinely available, since few hospitals have the resources to conduct regular audits of antimicrobial use.

The APEASE (Affordability, Practicability, Effectiveness and cost-effectiveness, Acceptability, Side-effects/safety, and Equity) criteria for designing interventions provides a framework within which researchers can comprehensively consider the context-specific suitability of an intervention.[23] It has been demonstrated repeatedly within the field in which it was developed to assess the suitability of behaviour change intervention functions.[24–27] It also provides the potential to be a useful tool when considering the complexities of identifying a suitable standardized antibiotic consumption surveillance methodology for the high-income secondary care setting.

This review will therefore study attempts to quantify total systemic antibiotic use, appropriate antibiotic use and inappropriate use in high income settings in secondary care. This review will provide a summary of the ways in which antibiotic use can be measured alongside a narrative about the practicality of employing these metrics in secondary care based on an adaptation of the APEASE framework.

OBJECTIVES

For peer review only - http://bmjopen.bmj.com/site/about/guidelines.xhtml

The aim of this review is to provide an outline of the methodologies to quantify antibiotic usage among in-patients in secondary care. The objectives are:

- To identify the data sources and metrics which have most frequently been used to represent antibiotic usage in secondary care.
- To assess the suitability of antibiotic use surveillance methodologies for use in routine surveillance of antibiotic usage based on the APEASE criteria.
- To document the variation in definitions and estimates of appropriate and inappropriate antibiotic usage.

METHODS

The following methods are based on the framework provided by the 'Preferred Reporting Items for Systematic review and Meta-Analysis Protocols (PRISMA-P) checklist.[28] This review will begin in August 2018 and we aim to complete the review by September 2019.

Eligibility Criteria

Types of Studies

Study inclusion criteria:

- Studies which quantify antibiotic usage in at least 100 adult patients (>17 years old) among in-patients in secondary care will be included.
- This review will include studies in the adult inpatient, high-income secondary care setting as it is expected that quantities of antibiotic use and patterns of prescribing in non-adult inpatients is likely to be significantly different to those in adults.
- Studies which focus on antibiotic usage among individuals with specific conditions or specific demographic groups will be included.

For peer review only

- Those studies which do not provide enough information for a complete assessment using the APEASE criteria will be included in the final review, but excluded from the APEASE criteria evaluation.
- This review will therefore include a range of study designs which are based on both observation and intervention, and which have attempted to estimate antibiotic usage in secondary care.

Study exclusion criteria:

- This review will not include trials of antibiotic therapies in secondary care or reviews of the literature.
- Articles which describe a methodology for measuring antibiotic usage and which do not attempt to implement it to estimate antibiotic usage will be excluded.
- Articles which are not written in English-language and are based on non-human participants will be excluded.
- If the number of adult in-patients included in the study is not stated, the article will be excluded from the review.

Types of Participants

The study must be based on dispensing, prescribing, survey/audit or sales data collected from the in-patient secondary care setting. It is expected that hospitals in high-income settings will be very different to those in low-resource settings in both the availability of data and resources available for stewardship, therefore this review will focus on hospitals in high-income countries as classified by the World Bank only (**Table S1**).[29] When the country is not classified in the World Bank lending groups, the article will be included if the gross national income (GNI) per capita is 12,056 USD or more. Articles must provide data which quantifies antibiotic usage among adult patients (>17-years old).

Types of Interventions

For peer review only

This review will consider all attempts to measure antibiotic usage among at least 100 patients in the hospital in-patient setting.

Types of Comparators

There are no comparison groups for this review as it is an exploration to identify the methodologies of how antibiotic usage has been measured in secondary care, and how this has been used to define and estimate appropriate and inappropriate antibiotic usage.

Types of Outcome measures

Articles which report a measure of antibiotic usage in hospital will be included in this review.

Primary Outcomes

- Data source used to represent antibiotic usage.
- Metrics which attempt to quantify antibiotic usage.
- An analysis of the suitability of existing metrics of antibiotic usage for use in routine practice to monitor antibiotic usage based on an adaptation of the APEASE framework (detailed in *Data Synthesis*).[23]

Secondary Outcomes

- Quantitative definitions of appropriate and inappropriate antibiotic usage.
- Comparison of the definitions of appropriate and inappropriate prescribing in secondary care.
- Description of variations in estimates of appropriate and inappropriate prescribing in secondary care.

Information Sources

Electronic Searches

For peer review only

The following databases will be searched for relevant articles:

- Medline (Ovid);
- Embase (Ovid);
- Cumulative Index to Nursing and Allied Health Literature (CINAHL);
- Cochrane Central Register of Controlled Trials;

The search strategy will be developed in Medline and then adapted appropriately for use in each database. The search will be conducted across all databases on 2nd August 2018.

Website Searches

In order to capture reports from the grey literature, the websites of relevant organisations will be searched (**Table S2**). The list of organisations expands on a list originally conceived by *Benic et. al* based on discussion between SP, AJ and LS.[30] This review aims to consider the suitability of existing metrics of antibiotic usage in routine hospital surveillance of antibiotic usage; therefore it is essential that approaches to do this on a large scale such as through National Action Plans, which are often published in the grey literature, should be captured in our search. This search will be conducted in February 2019, the specific dates of which will be reported in the final review.

Search Strategy

The provisional search strategy is adapted from the work of *Benic et. al* based on four concepts: antibiotics; utilisation; measure; hospital (**Table 1**).[30] The search strategy will be established in MEDLINE and then adapted to the other 3 databases included in the search. No restrictions will be placed on the search to maximize sensitivity.

Table 1. Medline (Ovid) provisional search terms.

Search Concept	Search Terms

For peer review only - http://bmjopen.bmj.com/site/about/guidelines.xhtml

**Antibiotic**	1. Anti-Bacterial Agents/ad, dt, sd, tu, th, ut [Administration & Dosage, Drug Therapy, Supply & Distribution, Therapeutic Use, Therapy, Utilization]
	2. Antibiotic Prophylaxis/ec, mt, sn, td, ut [Economics, Methods, Statistics & Numerical Data, Trends, Utilization]
	3. (anti?biotic? or anti?microbial? or anti?bacterial?).ab,ti.
	4. 1 or 2 or 3
**Utilisation**	5. Drug Prescriptions/
	6. Drug Utilization/
	7. "Drug Utilization Review"/cl, ec, mt, st, sn, td, ut [Classification, Economics, Methods, Standards, Statistics & Numerical Data, Trends, Utilization]
	8. ((anti?biotic? or anti?microbial?) adj3 (prescri* or consumption or utili?ation or usage or "use" or dispens* or sale?)).ab,ti.
	9. 5 or 6 or 7 or 8
**Measurement**	10. BENCHMARKING/cl, ec, mt, st, sn, td, ut [Classification, Economics, Methods, Standards, Statistics & Numerical Data, Trends, Utilization]
	11. (intervention adj5 (prescri* or stewardship or "use" or utili?ation or usage or consumption)).ab,ti
	12. ((anti?biotic? or anti?microbial?) adj4 (estimat* or quanitf* or metric? or monitor* or surveillance or prevalence or survey or audit)).ab,ti.
	13. (electronic prescri* or e?prescri*).ab,ti.
	14. 10 or 11 or 12 or 13
**Secondary Care**	15. Secondary Care/
	16. Hospitals/
	17. hospital*.ab,ti.
	18. 15 or 16 or 17

For peer review only

	19. 4 and 9 and 14 and 18
**Legend.** .**ab**, abstract; **ti**., title; **adj3**, indicates two words next to each other in any order with up to 2 words in between; /, indicates a Medical Subject Heading (MeSH); *****, denotes any truncation; **?**, denotes one character or no character. All other abbreviations are elaborated upon in [ ] brackets in the table.	

Study Records

Data Management

The articles will be uploaded from the relevant database to *Mendeley* reference manager software. The reports will be exported to the web-based, *Evidence for Policy and Practice Information (EPPI) Reviewer 4* systematic review software which will be used to remove duplicate articles. Each stage of the screening process will be carried out in *EPPI Reviewer 4* using a standardised form of article eligibility. The online form for article eligibility will be piloted before the start of the screening process and amended as required to ensure it is fit for purpose. The data will be extracted and entered into a standardised, piloted, pre-created online data extraction form in *EPPI Reviewer 4* in duplicate by two independent reviewers (SP and AJ).

Selection Process

The unique articles captured by the search will undergo a title pre-screening by a single reviewer (SP). The remaining articles will then be screened based on title and abstract by two independent reviewers in duplicate (SP and AJ) ; when study eligibility is not mutually agreed upon or is ambiguous from the title and abstract only, the article will be admitted into the full-text review stage. The selected articles will undergo full-text review by two independent reviewers in duplicate (SP and AJ). Any disagreements between authors at any stage will be resolved through a third reviewer (LS). The reasons

For peer review only

for the exclusion of these studies from this review will be documented for reference and the study selection process will be presented as a PRISMA flow diagram.[28]

Data Collection Process

The data will be collected in duplicate by two independent reviewers (SP and AJ) from articles selected for inclusion in the review. The data will be entered into pre-created, piloted forms on EPPI Reviewer 4: systematic review software. Any disagreement will be resolved through consultation with a third author (LS).

Data Items

We will extract data on:

- **Study** (study design, country, population, service setting such as size of hospital, objective).
- **Data type used to describe antibiotic usage** (data source, method of collection, frequency of the data collection, reason for data collection e.g. national surveillance or research, who collected the data, level of detail in the data and data type).
- **Metrics used to measure antibiotic usage** (numerator, denominator, units (such as per admission), level at which the metric is presented (such as by specialty).
- **Criteria used to define appropriate and inappropriate antibiotic usage**.
- **Data type used to quantify appropriate and inappropriate antibiotic usage** (data source, method of collection, level of detail in the data and data type).
- **Quantitative estimates of appropriate and inappropriate antibiotic usage** (numerator, denominator, units (such as per admission), level at which the metric is presented (such as by specialty) and what the metric is currently used for (such as national or hospital-level surveillance)).

For peer review only

- **Study quality and selection bias of those studies which provide an estimate of appropriate and inappropriate antibiotic usage** (outlined below under *Assessment of Risk of Bias*).

Outcomes Prioritisation

The main outcome of this review is a description of the data sources and metrics which have been used to measure antibiotic usage and to define appropriate and inappropriate antibiotic usage in hospitals. This will include a description of the teams most frequently involved in this type of work in order to identify those groups who are often facilitators of this surveillance.

This will then be used to consider each surveillance method within an adaptation of the APEASE framework to assess the practicality of each method and metric for use in routine surveillance of antibiotic usage; this method is detailed in '*Data synthesis'*.[23] This aims to inform the methodology of sustainable antibiotic usage surveillance programmes. We will also document if it is stated that the metric is currently being routinely used for any purpose such as national level surveillance. This will identify reasons why antibiotic usage surveillance is already being implemented and combined with methodological details will reveal programmes where existing infrastructure may be extended for use to routinely monitor antibiotic usage within hospitals. If the metric has been used to define and estimate (in)appropriate antibiotic usage, this will be used to explore the variation in the definitions and estimates of inappropriate antibiotic usage in hospitals. It is anticipated that such estimations will have been made using a multitude of methodologies and measures. The extent to which quantitative estimates of inappropriate prescribing can be directly compared may therefore be limited.

Assessment of Risk of Bias in Individual Studies

All metrics of antibiotic consumption included in the final review must have been utilized in the secondary care setting to capture the antibiotic usage data for at least 100 patients. This is to ensure some level of demonstration of the feasibility of implementing the measures. For these descriptive purposes, it is not appropriate to use a risk of bias framework.

For peer review only - http://bmjopen.bmj.com/site/about/guidelines.xhtml

For studies included in the full-text review stage and which attempt to make a quantitative estimate of appropriate or inappropriate antibiotic usage in the secondary care setting, the Newcastle-Ottawa scale to assess study quality will be used to determine the inclusion of the estimate in the review.[31]

Data synthesis

The data will undergo a descriptive synthesis to provide insight into the methodologies to implement antibiotic usage metrics in secondary care. We will record the frequency of use of different data sources by level (individual to national) and by source (such as electronic prescribing or audits). This will examine the type of data which is currently being used to monitor antibiotic usage and consequently the level detail in which we are able to examine prescribing currently. We will also identify the frequency of use of each metric to measure: volume, type, duration, stewardship activity and prevalence/incidence of antibiotic use. As well as identify the level at which these metrics are most frequently presented such as by specialty or nationally. The teams involved in these studies to collect and analyse the data will also be recorded.

An adaptation of the APEASE framework will be used to assess the feasibility of metrics for use in routine monitoring of antibiotic usage. The APEASE framework was designed to aid the context-specific design and selection of interventions to promote health behaviour change.[23] The framework acronym is elaborated to: a – affordability; p – practicability; e – effectiveness and cost-effectiveness; a – acceptability; s – side-effects/safety; e – equity. This original framework will be adapted to inform the establishment of a consensus to monitor antibiotic usage in the high-income secondary care setting [**Table 2**].

Table 2. An adaptation of the APEASE framework to assess the feasibility of metrics for use in routine surveillance.

For peer review only

**A**	[Affordability] Resources required to sustainably implement the metric. For example, person time to audit / new system roll-out.
**P**	[Practicability] Can the metric be delivered? Is there sustainable access to data and resources?
**E**	[Effectiveness] Does the surveillance method capture sufficient data?
**A**	[Acceptability] Does it represent relevant data to monitor antibiotic usage and appropriateness of antibiotic usage?
**S**	[Side-effects] Bias of metrics
**E**	[Equity] Is it feasible for hospital trusts of varying level of resources to all implement the surveillance methodology?

This analysis can then be used to inform recommendations based on the available literature regarding a sustainable, conventional strategy to monitor antibiotic usage and the resources which would be required to practically implement such a surveillance strategy.

The variation between comparable hospital-level estimates of (in)appropriate antibiotic usage will be represented using a funnel plot of inappropriate prescribing estimates against patient numbers and 95% confidence intervals.

Meta-bias(es)

The systematic review will be conducted in line with this protocol and any aspects of the method which deviate from the original protocol will be reported in the final review. Only articles written in English-language will be included in this review; this is due to the language spoken by the authors.

Confidence in Cumulative Evidence

This is a descriptive review to consider the methodologies which have been used to measure antibiotic usage and to define appropriate and inappropriate antibiotic prescribing in hospitals. The methodologies

For peer review only

will be considered in terms of the APEASE criteria and it is therefore not necessary to consider the quality of quantitative evidence for all studies. For observational studies which attempt to make a quantitative estimate of appropriate or inappropriate antibiotic usage in the secondary care setting, the Newcastle-Ottawa scale to assess risk of bias will be used to determine the inclusion of the estimate in the review.[31]

Patient and Public Involvement

Patients and members of the public were not involved in the development of this protocol.

Contributors

The study was conceived by SP, SH and LS. SP developed the eligibility criteria, search strategy, risk of bias assessment and the data extraction plans with guidance from LS. SP, AJ and LS developed the website search list. All authors contributed to and reviewed the final manuscript.

Acknowledgements

The authors acknowledge the valuable technical guidance offered by Suvi Härmälä in the construction of the protocol.

Funding

This review was funded by the Economic and Social Research Council (ESRC); grant number: ES/P000592/1 awarded to SP. The ESRC was not involved in the development of this protocol.

Patient Consent

Not required.

Ethics Approval

Ethical approval is not required for this study.

For peer review only

CONFLICTS OF INTEREST

The authors declare no conflicts of interest.

BIBLIOGRAPHY

1. O'Neill, J. *TACKLING DRUG-RESISTANT INFECTIONS GLOBALLY: FINAL REPORT AND RECOMMENDATIONS THE REVIEW ON ANTIMICROBIAL RESISTANCE*. (2016).

2. Goossens, H., Ferech, M., Vander Stichele, R. & Elseviers, M. Outpatient antibiotic use in Europe and association with resistance: a cross-national database study. *Lancet* **365,** 579–587 (2005).

3. Costelloe, C., Metcalfe, C., Lovering, A., Mant, D. & Hay, A. D. Effect of antibiotic prescribing in primary care on antimicrobial resistance in individual patients: systematic review and meta-analysis. *BMJ* **340,** c2096 (2010).

4. World Health Organization. *WHO Report on Surveillance of Antibiotic Consumption*. (2018).

5. A., V. *et al.* Antimicrobial consumption and resistance in adult hospital inpatients in 53 countries: results of an internet-based global point prevalence survey. *Lancet Glob. Heal.* **6,** e619–e629 (2018).

6. Zanichelli, V. *et al.* Variation in antibiotic use among and within different settings: a systematic review. *J. Antimicrob. Chemother.* **73,** vi17-vi29 (2018).

7. Public Health England. English surveillance programme for antimicrobial utilisation and resistance (ESPAUR) report - GOV.UK. Available at: https://www.gov.uk/government/publications/english-surveillance-programme-antimicrobial-utilisation-and-resistance-espaur-report. (Accessed: 19th November 2018)

8. Australian Commission on Safety and Quality in Health Care. *Antimicrobial Use in Australian Hospitals 2016 annual report of the National Antimicrobial Utilisation Surveillance Program*.

For peer review only

(2018).

9. WHOCC. WHOCC - Definition and general considerations. Available at: https://www.whocc.no/ddd/definition_and_general_considera/. (Accessed: 24th January 2019)

10. SA Health. *Antimicrobial Use in Australian Hospitals: 2016 annual report of the National Antimicrobial Utilisation Surveillance Program Published by the Australian Commission on Safety and Quality in Health Care*. (2018).

11. Public Health England. *English Surveillance Programme for Antimicrobial Utilisation and Resistance (ESPAUR) 2017*. (2017).

12. Statens Serum Institute, National Veterinary Institute- Technical University of, Denmark & National Food Institute - Technical University of Denmark. *DANMAP 2017-Use of antimicrobial agents and occurrence of antimicrobial resistance in bacteria from food animals, food and humans in Denmark*. (2017).

13. Swedres-Svarm. SWEDRES |SVARM 2017 2 A report on Swedish Antibiotic Utilisation and Resistance in Human Medicine (Swedres) and Swedish Veterinary Antibiotic Resistance Monitoring (Svarm) Editors. (2017).

14. ECDC. About ECDC Point prevalence survey. Available at: https://ecdc.europa.eu/en/healthcare-associated-infections-acute-care-hospitals/facts/about. (Accessed: 31st January 2019)

15. NHS England. NHS England » Commissioning for Quality and Innovation (CQUIN) Guidance for 2017-2019. Available at: https://www.england.nhs.uk/publication/commissioning-for-quality-and-innovation-cquin-guidance-for-2017-2019/. (Accessed: 24th August 2018)

16. NHS England. NHS England » 2017/19 CQUIN. Available at: https://www.england.nhs.uk/nhs-standard-contract/cquin/cquin-17-19/. (Accessed: 13th December 2018)

For peer review only

17. Australian Commission on Safety and Quality in Health Care. *Antimicrobial prescribing practice in Australian hospitals*. (2018).

18. Nick, T. Drug errors cause appalling harm and deaths, says Hunt - BBC News. (2018). Available at: https://www.bbc.co.uk/news/health-43161929. (Accessed: 14th August 2018)

19. Australian Government Department of Health. Improving Access to Medicines – ePrescribing for safer medicines. Available at: http://www.health.gov.au/internet/budget/publishing.nsf/Content/budget2018-factsheet14.htm. (Accessed: 24th August 2018)

20. World Health Organization. *Global Action Plan on Antimicrobial Resistance*. (2015).

21. Monnier, A. A. *et al.* Quality indicators for responsible antibiotic use in the inpatient setting: a systematic review followed by an international multidisciplinary consensus procedure. *J. Antimicrob. Chemother.* **73,** vi30-vi39 (2018).

22. Stanić Benić, M. *et al.* Metrics for quantifying antibiotic use in the hospital setting: results from a systematic review and international multidisciplinary consensus procedure. *J. Antimicrob. Chemother.* **73,** vi50-vi58 (2018).

23. Michie, S., Atkins, L. & West, R. *The Behaviour Change Wheel Book - A Guide To Designing Interventions*. (2014).

24. Suntornsut, P. *et al.* Barriers and Recommended Interventions to Prevent Melioidosis in Northeast Thailand: A Focus Group Study Using the Behaviour Change Wheel. *PLoS Negl. Trop. Dis.* **10,** e0004823 (2016).

25. Sinnott, C. *et al.* Improving medication management in multimorbidity: development of the MultimorbiditY COllaborative Medication Review And DEcision Making (MY COMRADE)

For peer review only

intervention using the Behaviour Change Wheel. *Implement. Sci.* **10,** 132 (2015).

26. Cadogan, C. A. *et al.* Development of an intervention to improve appropriate polypharmacy in older people in primary care using a theory-based method. *BMC Health Serv. Res.* **16,** 661 (2016).

27. Mc Sharry, J., Murphy, P. J. & Byrne, M. Implementing international sexual counselling guidelines in hospital cardiac rehabilitation: development of the CHARMS intervention using the Behaviour Change Wheel. *Implement. Sci.* **11,** 134 (2016).

28. Shamseer, L. *et al.* Preferred reporting items for systematic review and meta-analysis protocols (PRISMA-P) 2015: elaboration and explanation. *BMJ* **350,** g7647 (2015).

29. The World Bank Group. World Bank Country and Lending Groups. Available at: https://datahelpdesk.worldbank.org/knowledgebase/articles/906519-world-bank-country-and-lending-groups.

30. Stanic Benic, M. *et al.* Metrics for quantifying antibiotic use in the hospital setting: results from a systematic review and international multidisciplinary consensus procedure. *J. Antimicrob. Chemother.* **73,** vi50-vi58 (2018).

31. Wells, G. *et al.* The Newcastle-Ottawa Scale (NOS) for assessing the quality of nonrandomised studies in meta-analyses. Available at: http://www.ohri.ca/programs/clinical_epidemiology/oxford.asp. (Accessed: 31st July 2018)

For peer review only

SUPPLEMENTARY MATERIALS

Table S1. ***World Bank Country Lending Groups Classification: High-Income Economies ($12,056 GNI per Capita or more)***

Accessed on: 23.08.18. Available at: https://datahelpdesk.worldbank.org/knowledgebase/articles/906519-world-bank-country-and-lending-groups

Countries				
Andorra	Germany	Oman	Antigua and Barbuda	Gibraltar
Palau	Argentina	Greece	Panama	Aruba
Greenland	Poland	Australia	Guam	Portugal
Austria	Hong Kong SAR, China	Puerto Rico	The Bahamas	Hungary
Qatar	Bahrain	Iceland	San Morino	Barbados
Ireland	Saudi Arabia	Belgium	Isle of Man	Seychelles
Bermuda	Israel	Singapore	British Virgin Islands	Italy
Sint Maarten (Dutch)	Brunei Darussalam	Japan	Slovak Republic	Canada
Korea Rep.	Slovenia	Cayman Islands	Kuwait	Spain
Channel Islands	Latvia	St. Kitts and Nevis	Chile	Liechtenstein
St. Martin (French)	Croatia	Lithuania	Sweden	Curaçao
Luxembourg	Switzerland	Cyprus	Macao SAR, China	Taiwan, China
Czech Republic	Malta	Trinidad and Tobago	Denmark	Monaco
Turks and Caicos Islands	Estonia	Netherlands	United Arab Emirates	Faroe Islands
New Caledonia	United Kingdom	Finland	New Zealand	United States
France	Northern Mariana Islands	Uruguay	French Polynesia	Norway
Virgin Islands (U.S.)				

For peer review only

Table S2. ***Website Search List***

Expanded upon from:

Stanic Benic, M. *et al.* Metrics for quantifying antibiotic use in the hospital setting: results from a systematic review and international multidisciplinary consensus procedure. *J. Antimicrob. Chemother.* **73,** vi50-vi58 (2018).

	Organisation	Website(s)
1	Africa CDC: African Union	https://au.int/en/africacdc
2	Agency for Healthcare Research and Quality	www.ahrq.gov/
3	Antibiotic Action	http://antibiotic-action.com/
4	Alliance for the Prudent Use of Antibiotics (APUA)	www.apua.org/
5	Australian Commission on Safety and Quality in Health Care | Antimicrobial Use and Resistance in Australia (AURA)	https://www.safetyandquality.gov.au/antimicrobial-use-and-resistance-in-australia/
6	Australian Government Department of Health	www.health.gov.au/
7	British Society for Antimicrobial Chemotherapy	http://www.bsac.org.uk/
8	British Infection Association	https://www.britishinfection.org/
9	Center for Disease Dynamics, Economics & Policy (CDDEP)	https://www.cddep.org/ https://resistancemap.cddep.org/AntibioticUse.php
10	Canadian Foundation for Healthcare Improvement	https://www.cfhi-fcass.ca/
11	Centers for Disease Control and Prevention (CDC)	https://www.cdc.gov/
12	Care Quality Commission	https://www.cqc.org.uk/
13	European Centre for Disease Control and Prevention (ECDC)	https://ecdc.europa.eu/en/home
14	European Society of Clinical Microbiology and Infectious Diseases (ESCMID)	https://www.escmid.org/
15	Healthcare Infection Association	https://www.britishinfection.org/
16	Institute for Healthcare Improvement: Australian Council for Safety and Quality Health Care	http://www.ihi.org/resources/Pages/OtherWebsites/AustralianCouncilforSafetyandQualityinHealthCare.aspx
17	International Society of Chemotherapy Infection and Cancer (ISC)	www.ischemo.org/
18	International Society of Infectious Diseases	www.isid.org/
19	National Institute for Health and Care Excellence (NICE)	https://www.nice.org.uk/
20	National Quality Measures Clearinghouse	https://www.qualitymeasures.ahrq.gov/
21	NHS Improvement	https://improvement.nhs.uk/
22	Public Health Agency of Canada	www.publichealth.gc.ca/

For peer review only

23	Public Health England	https://www.gov.uk/government/organisations/public-health-england<br>https://fingertips.phe.org.uk/profile/amr-local-indicators
24	Public Health Surveillance: New Zealand Ministry of Health & Institute of Environmental Science Research Ltd (ESR)	https://surv.esr.cri.nz/index.php
25	RAND Corporation	www.rand.org/
26	ReAct group	www.reactgroup.org/
27	Swedish Strategic Programme against Antibiotic Resistance (STRAMA)	www.strama.se
28	Transatlantic Task Force on Antimicrobial Resistance (TATFAR)	http://www.cdc.gov/drugresistance/tatfar/
29	US Food and Drug Administration	www.fda.gov/
30	World Health Organization	www.who.int/<br>http://www.afro.who.int/<br>https://www.paho.org/hq/<br>http://www.searo.who.int/en/<br>http://www.euro.who.int/en/home<br>http://www.emro.who.int/index.html<br>http://www.wpro.who.int/en/
31	Scottish Antimicrobial Prescribing Group	http://www.isdscotland.org/Health-Topics/Prescribing-and-Medicines/SAPG/

For peer review only

Reporting checklist for protocol of a systematic review.

Based on the PRISMA-P guidelines.

Instructions to authors

Complete this checklist by entering the page numbers from your manuscript where readers will find each of the items listed below.

Your article may not currently address all the items on the checklist. Please modify your text to include the missing information. If you are certain that an item does not apply, please write "n/a" and provide a short explanation.

Upload your completed checklist as an extra file when you submit to a journal.

In your methods section, say that you used the PRISMA-P reporting guidelines, and cite them as:

Moher D, Shamseer L, Clarke M, Ghersi D, Liberati A, Petticrew M, Shekelle P, Stewart LA. Preferred Reporting Items for Systematic Review and Meta-Analysis Protocols (PRISMA-P) 2015 statement. Syst Rev. 2015;4(1):1.

For peer review only

		Reporting Item	Page Number
Identification	#1a	Identify the report as a protocol of a systematic review	1
Update	#1b	If the protocol is for an update of a previous systematic review, identify as such	N/A

	#2	If registered, provide the name of the registry (such as PROSPERO) and registration number	2
Contact	#3a	Provide name, institutional affiliation, e-mail address of all protocol authors; provide physical mailing address of corresponding author	1
Contribution	#3b	Describe contributions of protocol authors and identify the guarantor of the review	1,18
	#4	If the protocol represents an amendment of a previously completed or published protocol, identify as such and list changes; otherwise, **state plan for documenting important protocol amendments**	17
Sources	#5a	Indicate sources of financial or other support for the review	18
Sponsor	#5b	Provide name for the review funder and / or sponsor	18
Role of sponsor or funder	#5c	Describe roles of funder(s), sponsor(s), and / or institution(s), if any, in developing the protocol	18
Rationale	#6	Describe the rationale for the review in the context of what is already known	4-7
Objectives	#7	Provide an explicit statement of the question(s) the review will address with reference to participants, interventions, comparators, and outcomes (PICO)	8+
Eligibility criteria	#8	Specify the study characteristics (such as PICO, study design, setting, time frame) and report characteristics (such	8-9

For peer review only

		as years considered, language, publication status) to be used as criteria for eligibility for the review	
Information sources	#9	Describe all intended information sources (such as electronic databases, contact with study authors, trial registers or other grey literature sources) with planned dates of coverage	10-11
Search strategy	#10	Present draft of search strategy to be used for at least one electronic database, including planned limits, such that it could be repeated	11-12
Study records - data management	#11a	Describe the mechanism(s) that will be used to manage records and data throughout the review	13
Study records - selection process	#11b	State the process that will be used for selecting studies (such as two independent reviewers) through each phase of the review (that is, screening, eligibility and inclusion in meta-analysis)	13
Study records - data collection process	#11c	Describe planned method of extracting data from reports (such as piloting forms, done independently, in duplicate), any processes for obtaining and confirming data from investigators	14
Data items	#12	List and define all variables for which data will be sought (such as PICO items, funding sources), any pre-planned data assumptions and simplifications	14

For peer review only

Outcomes and prioritization	#13	List and define all outcomes for which data will be sought, including prioritization of main and additional outcomes, with rationale	14-15
Risk of bias in individual studies	#14	Describe anticipated methods for assessing risk of bias of individual studies, including whether this will be done at the outcome or study level, or both; state how this information will be used in data synthesis	15-16
Data synthesis	#15a	Describe criteria under which study data will be quantitatively synthesised	16
	#15b	If data are appropriate for quantitative synthesis, describe planned summary measures, methods of handling data and methods of combining data from studies, including any planned exploration of consistency (such as I2, Kendall's τ)	17
	#15c	Describe any proposed additional analyses (such as sensitivity or subgroup analyses, meta-regression)	N/a
	#15d	If quantitative synthesis is not appropriate, describe the type of summary planned	16-17
Meta-bias(es)	#16	Specify any planned assessment of meta-bias(es) (such as publication bias across studies, selective reporting within studies)	17
Confidence in cumulative evidence	#17	Describe how the strength of the body of evidence will be assessed (such as GRADE)	17-18

For peer review only

The PRISMA-P checklist is distributed under the terms of the Creative Commons Attribution License CC-BY 4.0. This checklist can be completed online using https://www.goodreports.org/, a tool made by the EQUATOR Network in collaboration with Penelope.ai

For peer review only
